# Acute Modulation of Brain Connectivity in Parkinson Disease after Automatic Mechanical Peripheral Stimulation: A Pilot Study

**DOI:** 10.1371/journal.pone.0137977

**Published:** 2015-10-15

**Authors:** Carlo Cosimo Quattrocchi, Maria Francesca de Pandis, Claudia Piervincenzi, Manuela Galli, Jean Marc Melgari, Gaetano Salomone, Patrizio Sale, Carlo Augusto Mallio, Filippo Carducci, Fabrizio Stocchi

**Affiliations:** 1 Department of Medicine, Università Campus Bio-Medico di Roma, Rome, Italy; 2 Neuromotor Rehabilitation Unit, San Raffaele Hospital, Cassino, Italy; 3 Department of Physiology and Pharmacology, Neuroimaging Laboratory, Sapienza University, Rome, Italy; 4 Institute for Advanced Biomedical Technologies, University G. D’Annunzio Chieti-Pescara, Chieti, Italy; 5 Department of Electronics Information and Bioengineering, Politecnico di Milano, Milan, Italy; 6 Department of Neurology, Institute for Research and Medical Care, IRCCS San Raffaele, Rome, Italy; College of Mechatronics and Automation, National University of Defense Technology, CHINA

## Abstract

**Objective:**

The present study shows the results of a double-blind sham-controlled pilot trial to test whether measurable stimulus-specific functional connectivity changes exist after Automatic Mechanical Peripheral Stimulation (AMPS) in patients with idiopathic Parkinson Disease.

**Methods:**

Eleven patients (6 women and 5 men) with idiopathic Parkinson Disease underwent brain fMRI immediately before and after sham or effective AMPS. Resting state Functional Connectivity (RSFC) was assessed using the seed-ROI based analysis. Seed ROIs were positioned on basal ganglia, on primary sensory-motor cortices, on the supplementary motor areas and on the cerebellum. Individual differences for pre- and post-effective AMPS and pre- and post-sham condition were obtained and first entered in respective one-sample t-test analyses, to evaluate the mean effect of condition.

**Results:**

Effective AMPS, but not sham stimulation, induced increase of RSFC of the sensory motor cortex, nucleus striatum and cerebellum. Secondly, individual differences for both conditions were entered into paired group t-test analysis to rule out sub-threshold effects of sham stimulation, which showed stronger connectivity of the striatum nucleus with the right lateral occipital cortex and the cuneal cortex (max Z score 3.12) and with the right anterior temporal lobe (max Z score 3.42) and of the cerebellum with the right lateral occipital cortex and the right cerebellar cortex (max Z score 3.79).

**Conclusions:**

Our results suggest that effective AMPS acutely increases RSFC of brain regions involved in visuo-spatial and sensory-motor integration.

**Classification of Evidence:**

This study provides Class II evidence that automatic mechanical peripheral stimulation is effective in modulating brain functional connectivity of patients with Parkinson Disease at rest.

**Trial Registration:**

Clinical Trials.gov NCT01815281

## Introduction

Posture and gait disorders are among the most debilitating symptoms of Idiopatic Parkinson Disease. Particularly freezing of gait is defined as the inability to initiate gait or to manage narrow space- or direction-related challenges of gait, thus increasing the risk of falls [1,2] and reducing quality of life in Idiopatic Parkinson Disease patients [3]. Particularly, functional Magnetic resonance Imaging (fMRI) studies have shown that freezing in Parkinson’s Disease (PD) is related to dysfunction within fronto-parietal regions [4], within the pre-supplementary motor area and the anterior insula in response to concurrent cognitive and motor tasks [5], within fronto-parietal regions, basal ganglia (caudate nucleus, globus pallidus, subthalamic nucleus) and the mesencephalic locomotor region during performing a virtual reality task [6]. Moreover, gait imagery tasks have shown decreased activity in the supplementary motor area and increased activity in the mesencephalic locomotor region in PD patients with freezing, as compared to those without freezing [7,8]. The executive attention network and the visual network connectivity has been found to be significantly different in PD patients with freezing as compared to PD patients without freezing and controls [9]. Thus, there is currently strong evidence that alterations of the peripheral afferent inputs and/or their central processing alterations (i.e. sensory-motor integration) influence motor disability in patients with PD [10, 11].

Resting state functional MRI is currently widely used to study spontaneous fluctuations of the Blood Oxygen Level Dependent (BOLD) signal while the patient is at rest [12], thus also fitting for people who are unable or have difficulty in performing functional tasks [13]. High temporal coherence of the spontaneous BOLD signal fluctuation among different brain areas is interpreted as a measure of functional connectivity between those areas and has led to the identification of brain functional networks [14]. Analysis of functional brain networks and related connectivity measures has been obtained to explain non-motor [15–19] and motor [9, 20–25] deficits and to define their neural correlates in PD. These studies have shown alterations of brain connectivity in early-stage drug-naïve PD patients [22, 26] and in patients at advanced stage of the disease with cognitive [27] and severe motor [28] impairments.

To date, the effects of rehabilitation and exercise on brain activity have been poorly explored in patients with PD, but fMRI is considered of value to measure the effects of rehabilitation strategies in PD [29].

It has been reported that mechanical stimulation of the soles of the feet improves gait performance in PD [30–31]. Quantitative measures of gait analysis parameters have shown to increase step length and gait velocity together with autonomic sympathetic modulation in PD patients 24h after automatic mechanical peripheral stimulation (AMPS) [32]. More recently, PD patients have shown improvement of their ability to perform the Timed Up and Go Test after AMPS [33] and clinical benefits are maintained up to 10 days after the last treatment [34].

Thus, AMPS is emerging as a new and promising technique to improve posture and gait symptoms in PD, but mechanisms or neural correlates underlying its effects are currently unknown. The modulation of brain activity after AMPS has never been investigated. The aim of this work was to investigate whether and which measurable RSFC changes exist after AMPS in patients with Idiopathic Parkinson Disease.

## Materials and Methods

### Ethics Statements and study outcomes

**Fig 1:**
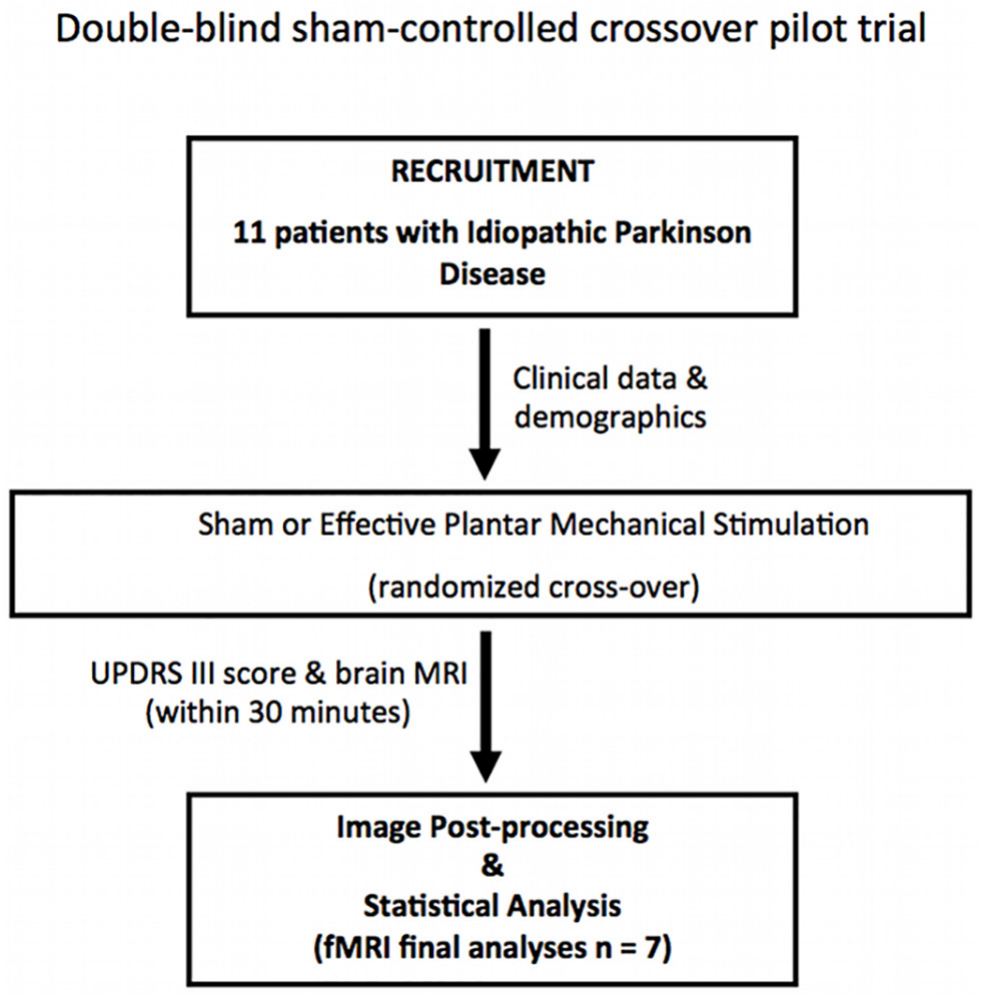
Flowchart of the experimental protocol of the pilot interventional study as approved by the local ethical committee.

The study was approved by the ethical committee of the Institute for Research and Medical Care, IRCCS San Raffaele, Rome, Italy (S1 Protocol; S2 Protocol). The experimental protocol was designed as a double-blind sham-controlled crossover pilot trial to test the acute modulation of brain functional connectivity in PD patients after AMPS (Fig 1) (ClinicalTrials.gov ID: NCT01815281; see S1 Clinical Trials Pilot). All procedures were explained, adequate understanding was tested and written informed consent was obtained from the participants in accordance with the declaration of Helsinki. Data were collected in compliance with GCP (Good Clinical Practice) and following the ALCOA (Attributable, Legible, Contemporaneous, Original and Accurate) algorithm. The TREND checklist was accomplished (S1 TREND Checklist).

The primary outcome of the study was to detect changes of brain functional connectivity after AMPS in patients with PD.

### Patients and clinical assessment

From September 15th to October 30th 2013, we investigated 11 patients (6 women and 5 men) with a diagnosis of PD according to the clinical diagnostic criteria of the United Kingdom Parkinson’s disease Society Brain Bank. Inclusion criteria were: 1) age of 45 years or older; 2) a Hoehn & Yahr (H&Y) stage equal or less than 3.0 in an ON state; 3) subjects able to walk autonomously or with minimal assistance for a distance of 10 meters in OFF state (12 h without antiparkinsonian treatment); 4) antiparkinsonian treatment at a stable and optimized daily dosage during the 4 weeks prior to the study; 5) absence of dopaminergic long-lasting residual effects. Exclusion criteria were: 1) Dementia established on the basis of the Mini-Mental State examination (corrected score < 27); 2) history or presence of peripheral sensory neuropathy; 3) any peripheral neurological or musculoskeletal conditions that may alter balance and/or gait; 4) lower limb injuries in the previous 6 months; 5) history of neurosurgery or orthopaedic surgery; 6) history of epilepsy; 7) any drug treatment that may alter cognitive and/or motor performance; 8) history of depression or other psychiatric disorders; 9) absolute contraindications for MRI; 10) evidence of small vessel ischemic disease or suggestive findings of secondary parkinsonism on morphological MRI images.

Patients underwent brain MR examination as outpatients and received clinical examination in a dedicated room beside the MRI magnet site. All patients were scanned twice in the same morning (between 8.30 am and 12.00 am), immediately before and < 30 min after sham or effective AMPS. Before and after each scanning session, motor performance was measured outside the scanner according to the Unified Parkinson's Disease Rating Scale (III) score by an experienced neurologist (G.S. or M.F.D.) who was blinded to the patients’ treatment status. To avoid potential residual effect between stimulations, the sham and the effective trial on the same patient were conducted with a time delay of at least 15 days. Patients were randomly selected to first undergo either effective AMPS (n = 6) or sham (n = 5) stimulation. All data were collected in OFF state (after a 12h withdrawal of anti-Parkinson medication). Motor severity was assessed by using the motor subscale of the Movement Disorders Society Unified Parkinson’s Disease Rating Scale (MDS-UPDRS part III).

### Automatic Mechanical Peripheral Stimulation Treatment

**Fig 2:**
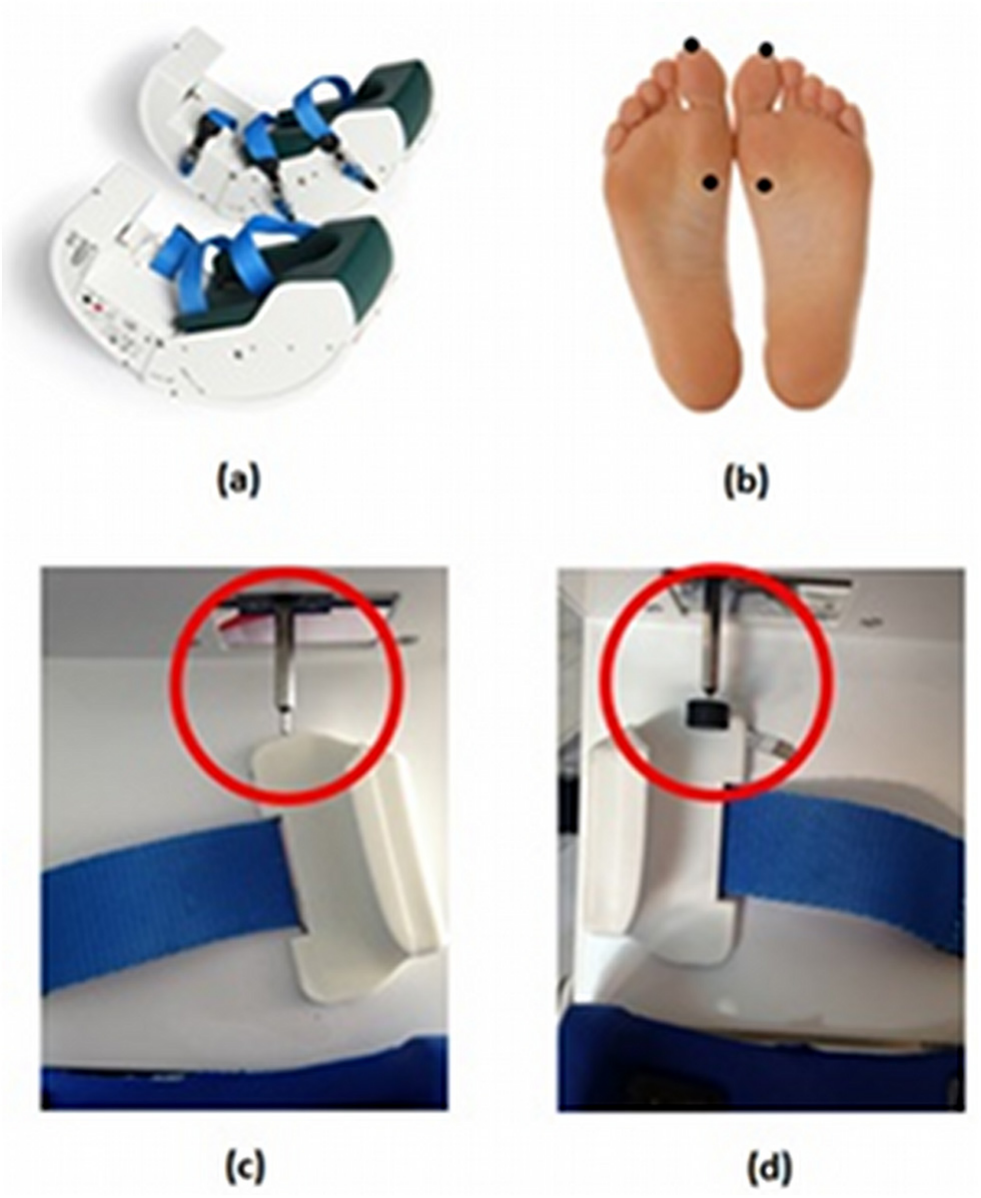
The device used for the AMPS treatment (a), the sites of feet stimulation (b), the effective AMPS (c), and the sham stimulation (d).

Effective and sham Automatic Mechanical Peripheral Stimulation (AMPS) trials were conducted by means of a dedicated electro-medical device (Gondola Medical Technologies SA, Switzerland) (Fig 2). The system consists in feet supports (left and right) (Fig 2A) with electrical motors that activate metallic stimulators having a rounded 2 mm tip; the motor-activated stimulators apply a mechanical pressure in two specific areas of each foot. The AMPS treatment consists in the application of a pressure via rounded stimulation tips in the four areas to be stimulated (two in each foot, corresponding to the head of the big toe and at the first metatarsal joint–Fig 2B and 2C). The pressure is applied in a range of 0.3–0.9 N/mm^2^ in each point, one after the other; the pressure of stimulation, within said range, is set for each subject upon appearance–during application of the stimulus–of the monosynaptic reflex in the Tibialis Anterior muscle, identified by detection of a liminaris contraction. Once the pressure value has been set using this procedure, the value is recorded to administer the AMPS. The treatment consists in 4 cycles, whereas cycle means the sequential stimulation of the 4 target areas for the duration of 6 seconds each, with no intervals in between; consequently a single cycle has duration of 24 seconds, while the overall treatment including four cycles lasts 96 seconds. The trials were conducted in an isolated room to keep the treatment blind to the clinicians and neuro-radiologists involved in the study [32].

The sham stimulation was provided using the same device utilized to give effective AMPS, with the same stimulation protocol and therapy cycle, applying in the same pressure stimulation points using the same steel stick for the AMPS stimulation. However, attached in the steel stick point was positioned a rigid plastic circle with a diameter (12mm); thanks to this, the induced pressure was hence lower and the surface contact bigger (Fig 2D). Sham stimulation does not trigger the reflex withdrawal of the stimulated foot and was used to eliminate confounding effects of brain activation induced by peripheral but not effective stimulation. No other procedures were conducted due to the full compliance of the patients to the interventions. We did not report any adverse event or unintended effect in each of the study conditions.

### fMRI data acquisition

Imaging data were acquired using a Siemens 1.5-T MAGNETOM Avanto (Siemens, Erlangen, Germany) whole body scanner equipped with a 12-element designed Head Matrix coil, as part of the standard system configuration. A morphological 3D-MPRAGE T1 weighted sequence was acquired to improve registration of functional images: TR = 1900ms, TE = 3.37ms, TI = 1100ms, flip angle = 15°, FOV = 256mm×192mm, NEX = 1, matrix = 256×192, 1.00×1.00 mm^2^ in-plane resolution, horizontal slices with slice thickness of 1.3 mm and no gap. Axial fluid attenuated inversion recovery (FLAIR) T2 weighted scan was used to exclude the presence of small vessel ischemic disease and other supra- or infra-tentorial brain lesions: TR 8000 ms, TE 102 ms, TI 3650 ms, matrix 256 x 256, FOV 26 x 30 cm, slice thickness 3 mm.

Whole brain functional scans were acquired in 25 contiguous axial slices approximately parallel to the anterior-posterior commissure plane with interleaved multi-slice T2* echo-planar imaging according to the following parameters: TR = 3.56 ms, TE = 50 ms, field of view = 22 cm, flip angle = 90°, voxel size = 3.4 × 3.4 × 3 mm, slice thickness = 3 mm, no inter-slice gap. For each participant, a total of 80 volumes during 4.50 min were acquired. fMRI scanning was carried out in darkness, and the participants were explicitly instructed to relax, think of nothing in particular, not to fall asleep and stay as much still as possible with their eyes closed. At the end of the MR examination, all participants were asked about their feelings during the scan and the tendency to sleep during the scanning. None of the subjects fell asleep or reported significant feelings during the scans.

### Data Pre-Processing and functional connectivity analysis

Single-subject preprocessing was carried out using FEAT (FMRI Expert Analysis Tool), Version 6.00, part of FSL v 5.0.4 (FMRIB’s Software Library http://fsl.fmrib.ox.ac.uk/fsl). Prestatistical processing consisted of motion correction using MCFLIRT [35], brain extraction using BET [36] and spatial smoothing using a Gaussian kernel of full-width at half-maximum (FWHM) of 8 mm. Large signal drifts (due to scanner instabilities or systemic physiological fluctuations) were attenuated by applying a high-pass filtering cut-off, set at 150 seconds [37–39]. Registration to high resolution structural and/or standard space images was carried out using FLIRT [35, 40]. EPI volumes were registered to the individual’s structural scan using FLIRT_BBR (Boundary-Based Registration) tool [41]. Registration from high resolution structural to standard space was then further refined using FNIRT nonlinear registration [42, 43].

Resting state functional connectivity (RSFC) analysis was carried out using a seed-ROI based approach [12, 44–46]. Based on previous evidence of local functional alterations of resting state fMRI measurements in Parkinson’s Disease patients [47], we explored the changes of functional connectivity with the supplementary motor area (SMA), primary sensory-motor cortices (as on the FSL implemented Harvard-Oxford Cortical Structural Atlas) [48], cerebellum (as on the MNI Structural Atlas), and basal ganglia (left and right nucleus striatum or left and right globus pallidus or left and right thalamus) [49]. Regions of Interest (ROIs) positioned on these areas were used as the seeds for resting state functional connectivity. Basal ganglia ROIs were labeled and calculated from each subject’s high resolution T1-weighted structural scan using FMRIB’s integrated registration and segmentation tool (FIRST) [50], part of FSL. To be more restrictive in ROIs segmentation, we chose an arbitrary inclusion threshold of 30% for the primary sensory-motor cortices and cerebellum, and of only 5% for the SMA (in an attempt to include both SMA proper and pre-SMA components, known to be altered from resting state studies conducted on patients with Parkinson’s Disease) [47, 51]. An expert neuroradiologist (CCQ) visually inspected the chosen ROIs in order to validate anatomical correspondence. EPI-to-standard registration parameters were inverted and used to report all seed-ROIs to individual subject space, applying both linear and non-linear transformation matrices from standard and high resolution space. Mean BOLD time series of each native space seed-ROI were obtained and used to define the reference time course, after which a correlation analysis between each reference time course and the signal time series in each voxel within the acquired whole-brain image set was computed.

Seeds of cerebro-spinal fluid (CSF) and white matter (WM) were also individually defined in the lateral ventricles and in the centrum semi-ovale on the functional EPI images, and their time courses were added, as non-interest covariate (nuisance), into the voxel-wise correlation analyses, to remove for non-neural contributions to the BOLD signal and enhance specificity [52].

Analyses were conducted both without and with inclusion of 6 head motion parameters into the voxel-wise correlation analysis to control for effects of transient head motion of patients on our results [53].

Z-score functional connectivity (FC) maps were generated for each subject by displaying all those voxels whose time series were correlated with the seed region (p<0.05). Maps of individual FC differences for pre- and post-effective AMPS (treatment) and pre- and post-sham conditions were initially obtained and entered in respective higher-level one-sample t-test analyses using FEAT (cluster level p<0.05 corrected for family wise error—FWE) to investigate for the mean effect of PMS and sham conditions on FC, separately. Then, a second higher-level analysis was conducted performing a paired T-test between maps of individual FC differences in effective and sham conditions. Higher level analyses were carried out using the FLAME (FMRIB's Local Analysis of Mixed Effects) mixed-effects model, stage 1 [54–56]. Z-statistic images were set using clusters determined by Z>2.3 and a corrected cluster significance threshold of p < 0.05 [57]. Anatomical localization of significant clusters was established according to the Harvard-Oxford cortical and subcortical structural atlases and the Juelich Histological Atlas included in the FSL (http://www.fmrib.ox.ac.uk/fsl/data/atlas-descriptions.html). Clinical scores before an after sham or effective AMPS were tested with the Wilcoxon-signed rank test for paired data by means of the SPSS software package 19.0.

## Results

### Patients

The 11 patients enrolled in the study were randomly assigned to a sham or effective AMPS according to the cross-over design (Fig 1). Patients showed a clinical akinesia/rigidity subtype in 72% (8/11) while a tremor subtype was present in the remaining 28% (3/11) of the patients. All the patients were right-handed and at least 12 h OFF medication. Demographic and clinical parameters are presented in Table 1 (S1 Database). The clinical evaluation before and after experimental sessions showed a statistically significant improvement of the UPDRS III scale (p < 0.001) after effective AMPS but not after sham stimulation (p = 0.87). The total Postural Instability Gait Disturbances (PIGD) UPDRS sub-score significantly improved after effective AMPS both with and without the inclusion of the 3.11 (freezing) score. However, the tremor and the rigidity sub-scores were not affected by effective AMPS. Sham stimulation did not induce significant modifications in any of the clinical measures. (Table 1)

**Table 1:** Demographic and clinical parameters of patients with Parkinson’s disease before and after effective AMPS and sham stimulation.

Parameter	Pre-Effective Stimulation mean ± SD	Post-Effective Stimulation mean ± SD	Effective Stimulation P-value	Pre-Sham Stimulation mean ± SD	Post-Sham Stimulation mean ± SD	Sham Stimulation P-value
Age (years)	62.27 ± 7.94 (range, 49–75)					
Gender (M/F)	5/6					
DD (years)	7.50 ± 2.22 (range, 4–10)					
H&Y stage	2.32 ± 0.25 (range, 2.00–2.50)					
MMSE	29.55 ± 0.82 (range, 28–30)					
Side (R/L)	7/4					
LEDD (mg/day)	789 ± 123 (range, 580–1000)					
UPDRS III OFF medication	30.55 ± 6.95^#^ 32.14 ± 7.58*	19.27 ± 6.36^#^ 21.14 ± 7.10*	**<0.001** ^#^ **0.016** *	27.27 ± 8.04^#^ 28.71 ± 9.69*	27.82 ± 7.21^#^ 29.57 ± 8.06*	0.868^#^ 0.860*
Tremor subscore^a^	2.91 ± 3.50^#^ 2.92 ± 4.11*	2.18 ± 2.44^#^ 1.57 ± 2.57*	0.578^#^ 0.704*	3.45 ± 3.27^#^ 3.14 ± 3.80*	3.73 ± 3.29^#^ 3.29 ± 3.82*	0.847^#^ 0.945*
PIGD subscore^b^	6.18 ± 3.49^#^ 7.43 ± 3.46*	3.09 ± 2.38^#^ 4.00 ± 2.45*	**0.025** ^#^ **0.053** *	4.55 ± 2.02^#^ 5.14 ± 1.95*	5.09 ± 2.38^#^ 5.71 ± 2.21*	0.569^#^ 0.618*
PIGD sub score with no freezing	5.36 ± 2.87^#^ 6.43 ± 2.88*	2.64 ± 2.06^#^ 3.43 ± 2.15*	**0.019** ^#^ **0.047** *	3.91 ± 1.76^#^ 4.43 ± 1.72*	4.27 ± 1.95^#^ 4.86 ± 1.95*	0.651^#^ 0.670*
Rigidity subscore^c^	6.36 ± 2.58^#^ 6.71 ± 3.09*	4.64 ± 2.87^#^ 5.43 ± 3.41*	0.153^#^ 0.474*	5.64 ± 2.20^#^ 5.86 ± 2.54*	5.45 ± 2.34^#^ 5.57 ± 2.37*	0.853^#^ 0.831*

Acronyms: M: male; F: female; DD: disease duration; H&Y: Hoehn and Yahr; MMSE: Mini-Mental State Examination; Side: Symtom-dominant side; LEDD: L-dopa equivalent daily dose; UPDRS: Unified Parkinson’s Disease Rating Scale; PIGD: Postural Instability Gait Disturbances

^#^ Data from the total group of patients (n = 11)

* Data from the group of patients eligible for fMRI analysis (n = 7).

^a^ Tremor sub score represents the sum of the UPDRS items in OFF condition: 3.15, 3.16, 3.17 e 3.18.

^b^ PIGD subscore represents the sum of the UPDRS items in OFF condition: 3.9, 3.10, 3.11, 3.12, 3.13, 3.14.

^c^ Rigidity subscore represents the sum of the UPDRS items in OFF condition: 3.3.

Statistically significant P-values (p < 0.05) are presented in bold.

Among the 11 patients, 7 (4 female, 3 men) were included for fMRI analyses of resting state functional connectivity. The exclusion of four participants was necessary because of the following reasons: one patient showed a large arachnoid cyst that caused anatomical distortion; three patients had at least one fMRI session with movement artefacts that significantly flawed data pre-processing and impeded intra- and inter-subject correction and registration. The 7 patients included in the fMRI study showed a clinical akinesia/rigidity subtype in 86% (6/7) while a tremor subtype was present in the remaining 14% (1/7) of the patients. In this subgroup of patients included for resting state functional connectivity, the effective AMPS consistently improved the UPDRS III score and the PIGD sub-score (with or without freezing) whereas there were not significant changes of the tremor and rigidity sub-scores.

### Changes of Functional Connectivity in the Resting State

**Fig 3:**
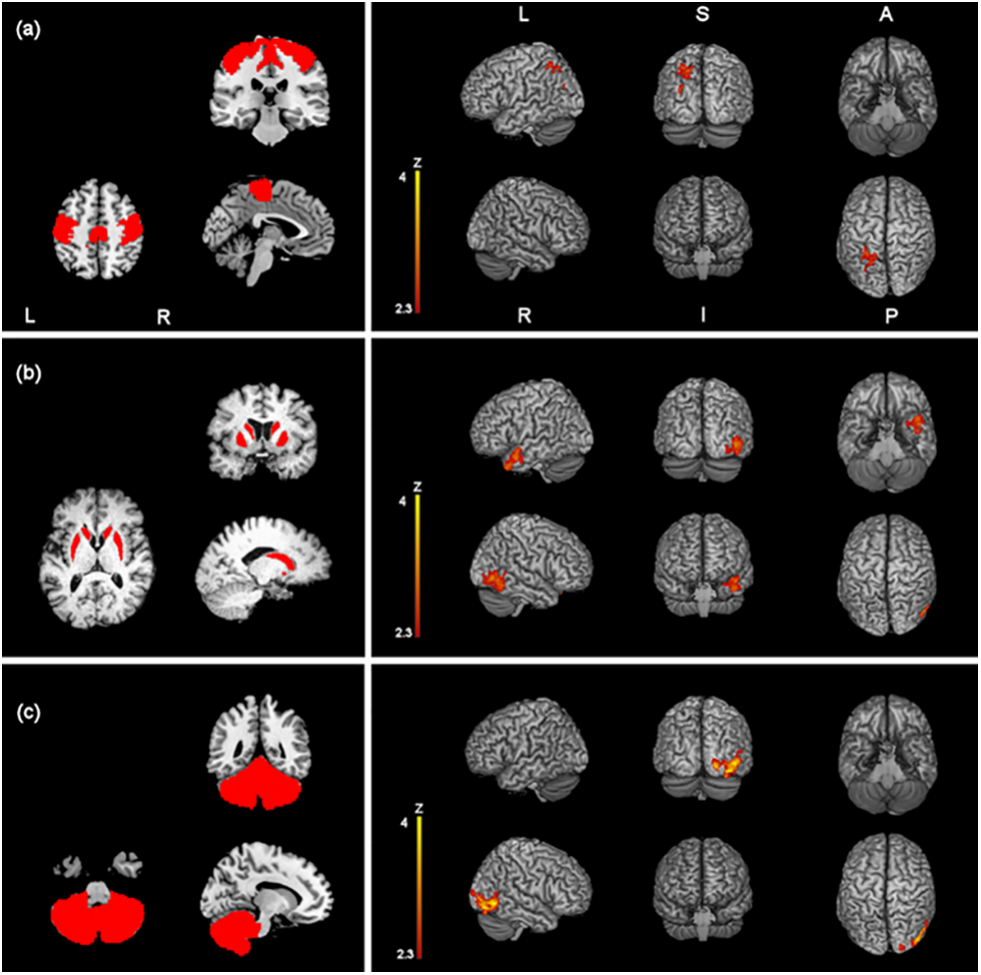
Z-statistic images showing clusters of significantly increased RSFC (p < 0.05, cluster-level FWE corrected) after one session of effective AMPS of the primary sensory motor cortex (a), the nuclei striati (c) and the cerebellum (d), overlaid onto a MNI-registered anatomical 3D-T1 volume. Seed regions of interest are red-coloured in the panels on the left. MNI coordinates (x, y, z) of the maximal Z-scores are presented in Table 2. Coronal and axial views follow the neurological convention.

One sample t-test analyses showed significant (p<0.05, FWE corrected) differences of RSFC with the selected seed regions following effective AMPS but not on sham conditions. Group differences were always oriented toward a significant increase of connectivity. Reduction of connectivity was not found with any of the seed regions. The primary sensory-motor cortex showed significantly stronger connectivity with the left superior parietal lobule and the left lateral occipital cortex (superior division) (t-tests) (Table 2 and Fig 3). The second higher level analysis to test the specific changes induced by the effective stimulation after ruling out sub-threshold effects of sham stimulation did not show significant changes of connectivity (mixed model).

**Table 2:** Local maxima in clusters of significantly (p < 0.05, FWE corrected) higher resting-state temporal correlation with the BOLD signal of the seed-ROIs after one session of AMPS.

Pre- vs. Effective AMPS	Cluster location	Cluster size (voxels)	MNI x	MNI y	MNI z	Z score
Primary Sensory-Motor Cortex	Left Superior Parietal Lobule	709	-26	-54	48	3.53
	Left Lateral Occipital Cortex					
Nucleus Striatus	Right Lateral Occipital Cortex, inferior	698	32	-72	-10	3.25
	Right Occipital Fusiform Gyrus					
	Right Temporal Pole	649	-42	8	-22	3.15
Cerebellum	Right Lateral Occipital Cortex	846	42	-80	-12	3.90
	Right Occipital fusiform gyrus					

**Fig 4:**
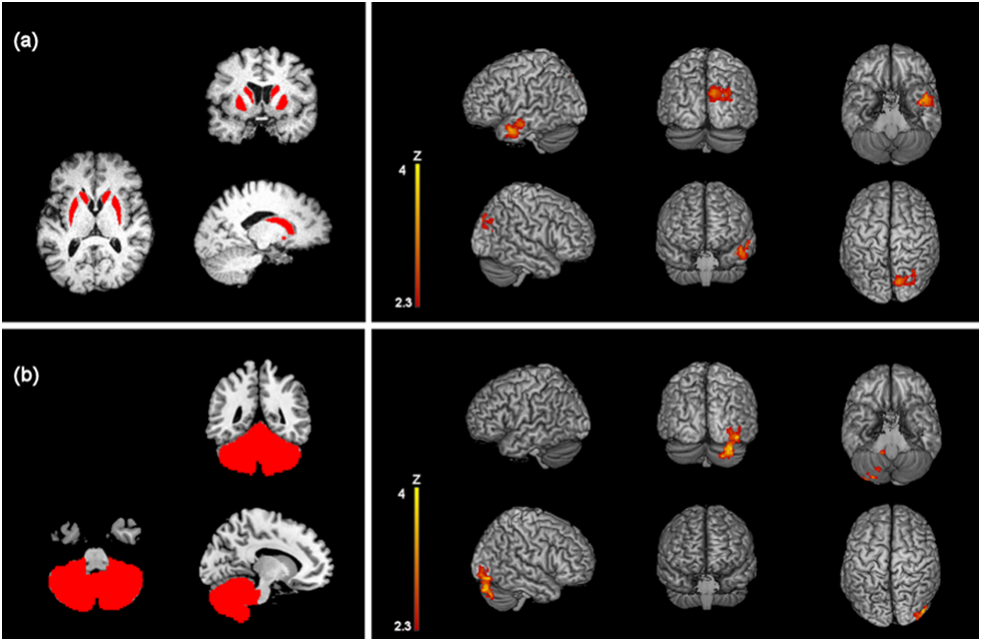
Z-statistic images showing clusters of significantly increased RSFC (p < 0.05, cluster-level FWE corrected) in the effective AMPS vs. sham stimulation for the nuclei striati (a) and the cerebellum (b). Seed regions of interest are red-coloured in the panels on the left. MNI coordinates (x, y, z) of the maximal Z-scores are presented in Table 3. Coronal and axial views follow the neurological convention.

The nuclei striati demonstrated significant increase of connectivity with a region that included the right lateral occipital cortex (inferior division) and the right occipital fusiform gyrus after effective but not after sham stimulation (t-tests) (Table 2 and Fig 3). The second higher level analysis confirmed significant increase of connectivity of the nucleus striatum with the right lateral occipital cortex and the cuneal cortex (Table 3 and Fig 4) (mixed model). Moreover, both t-test and the mixed model showed increased functional connectivity at rest between the nuclei striati and the right temporal pole (Tables 2 and 3, Figs 3 and 4).

**Table 3:** Local maxima in clusters of significantly (p < 0.05, FWE corrected) stronger connectivity in the effective AMPS vs. sham stimulation.

Effective vs. Sham	Cluster location	Cluster size (voxels)	MNI x	MNI y	MNI z	Z score
Nucleus Striatus	Right Lateral Occipital Cortex	558	12	-84	32	3.12
	Cuneal Cortex					
	Right Temporal Pole	637	-48	4	-24	3.42
Cerebellum	Right Lateral Occipital Cortex	857	46	-78	-12	3.60
	Right Crus I		34	-84	-30	3.79

The cerebellum showed significantly stronger connectivity with the right lateral occipital cortex and the right occipital fusiform gyrus after effective but not after sham stimulation (Table 2 and Fig 3). The second level analysis confirmed significantly higher connectivity induced by the effective stimulation with the right cerebellar cortex crus I (i.e. intraregional connectivity) and the right lateral occipital cortex (Table 3 and Fig 4).

**Fig 5:**
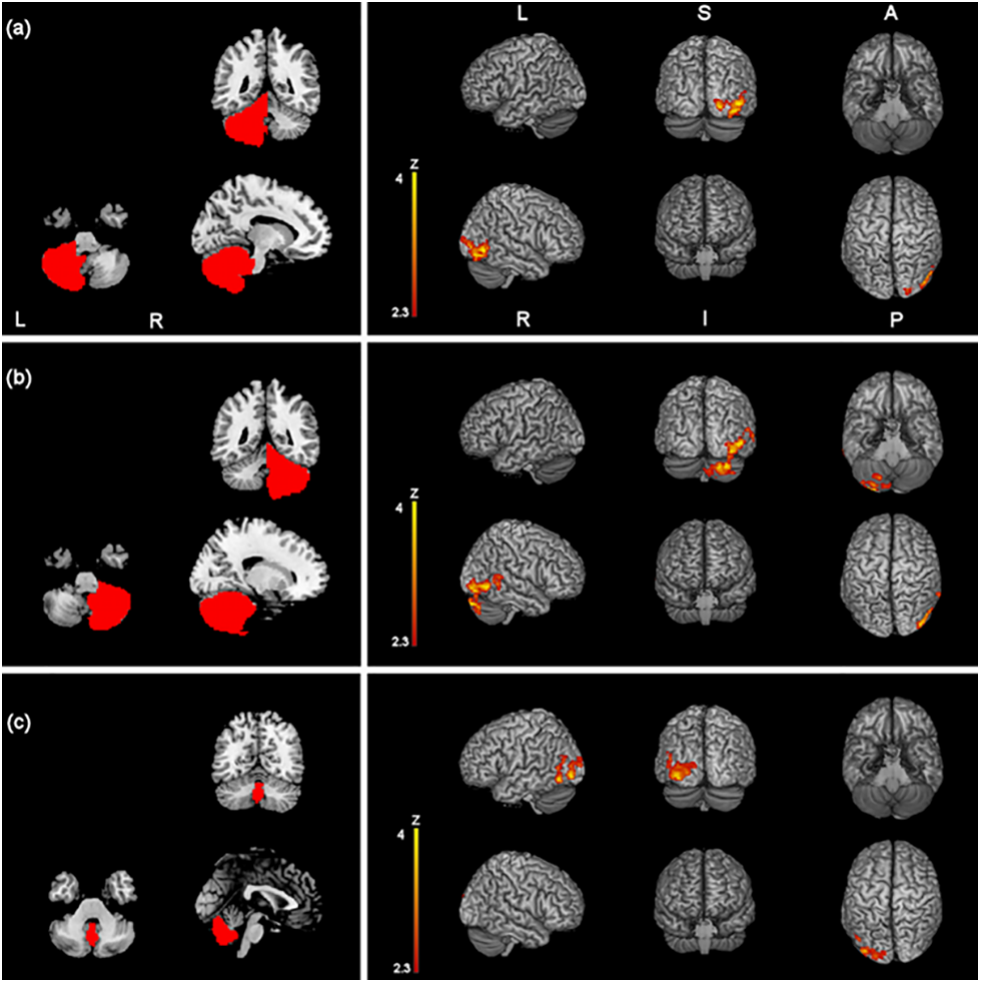
Z-statistic images showing clusters of significantly increased RSFC (p < 0.05, cluster-level FWE corrected) after one session of effective AMPS and in the effective vs. sham stimulation for the right cerebellar hemisphere (a), the left cerebellar hemisphere (b) and the vermis (c). Seed regions of interest are red-coloured in the panels on the left. MNI coordinates (x, y, z) of the maximal Z-scores are presented in Table 4. Coronal and axial views follow the neurological convention.

A further analysis that comprised the cerebellum subregions vermis, right hemisphere and left hemisphere confirmed increase of functional connectivity both with first-level (t-tests) and second-level (mixed model) analyses after effective stimulation. Vermis showed increase of connectivity with the left lateral occipital cortex and the left fusiform gyrus. Left and right cerebellar hemisphere considered separately both showed increase of connectivity with the right lateral occipital cortex and the right cerebellum crus I (Table 4 and Fig 5).

**Table 4:** Local maxima in clusters of significantly (p < 0.05, FWE corrected) stronger connectivity with cerebellum subregions (vermis, right cerebellum hemisphere, left cerebellum hemisphere). a. higher temporal correlation after one session of AMPS (pre- vs. post-effective stimulation); b. effective vs. sham plantar stimulation.

**Pre- vs. Effective AMPS**	**Cluster location**	**Cluster size (voxels)**	**MNI x**	**MNI y**	**MNI z**	**Z score**
Vermis	Left Lateral Occipital Cortex Left Fusiform gyrus	1158	-44	-70	-10	3.84
Right Cerebellum Hemisphere	Right Lateral Occipital Cortex	1331	52	-62	-6	3.94
	Right Crus I		34	-74	-34	3.88
Left Cerebellum Hemisphere	Right Lateral Occipital Cortex	797	22	-86	-6	3.95
**Effective vs. Sham**	**Cluster location**	**Cluster size (voxels)**	**MNI x**	**MNI y**	**MNI z**	**Z score**
Vermis	Left Lateral Occipital Cortex	753	-40	-88	-4	3.27
Right Cerebellum Hemisphere	Right Lateral Occipital Cortex	584	44	-86	-12	3.33
	Right Crus I		34	-84	-30	3.90
Left Cerebellum Hemisphere	Right Lateral Occipital Cortex Right Fusiform gyrus	951	46	-76	-12	3.64
	Right Crus I		36	-80	-30	3.75

The supplementary motor area, globi pallidi and thalami did not demonstrate above threshold changes of functional connectivity both on effective and sham conditions.

Results (Tables and Figures) obtained without inclusion of the 6 head motion parameters as nuisance variables are presented as Supplementary file (S1 Results).

## Discussion

In the current study, we explored the acute modulation of the resting state functional connectivity following one session of automatic mechanical peripheral stimulation in patients with Parkinson’s disease. Due to their established role in the pathophysiology of Parkinson Disease, we chose the sensory-motor cortex, the supplementary motor area, the basal ganglia and the cerebellum as “seeds” to investigate effects on the brain functional connectivity at rest [25, 58–60].

Our results indicate that the pressure controlled mechanical stimulation on two specific points of both feet increases brain functional connectivity at rest with the selected regions and that the effect is stable with the nucleus striatum and the cerebellum after inclusion of sub-threshold not significant effects following sham stimulation. The recruitment of a constellation of specific brain regions and the enhancement of their functional connectivity dynamics might be the basis for the acute improvement of posture and gait symptoms observed in Parkinson’s Disease patients after effective AMPS [32].

### Primary Sensory-Motor Cortex Functional Connectivity

Effective AMPS acutely induced consistent increase of at rest functional connectivity of the primary sensory-motor cortices with the left superior parietal lobule and the left lateral occipital cortex. These areas are known to be involved in sensory-motor and visuo-spatial integration and processing [61]. Particularly, the left primary motor cortex, the left pre-SMA and the left superior parietal lobule have shown significantly convergent difference of neural activity in Parkinson’s Disease OFF patients against healthy controls on a recent meta-analysis of functional neuroimaging studies on the motor control of Parkinson’s Disease [62]. In this respect, as compared with inconsistency on the direction of differences of other fronto-parietal areas, the left SPL consistently shows increased BOLD signal as compared to controls during the execution of different externally specified motor-tasks conducted on Parkinson’s Disease OFF patients, thus suggesting that SPL plays a compensatory role that is not dependent on the motor task in Parkinson’s Disease patients. Indeed, the increased functional connectivity that we observed after effective plantar stimulation also suggests that SPL activity may be modulated by external peripheral stimuli. It is striking that, together with SPL, also the lateral occipital cortex (LOC) showed a stronger functional connectivity after effective stimulation. The LOC plays a well-known role on object recognition [63–64], is included in the patterns of brain atrophy in Parkinson’s Disease patients with freezing of gait [65] and, as such, the modulation of its connectivity after peripheral stimulation might support the hypothesis of a crucial role of parietal regions on visuo-spatial integration in patients with Parkinson’s Disease.

### Basal Ganglia Functional Connectivity

At the level of the basal ganglia, we observed a significant increase of RSFC of the nucleus striatum but not of the thalami and the globi pallidi after effective AMPS. The regions of increased coherence with the nucleus striatum comprised the right occipital fusiform gyrus and the right lateral occipital cortex. These areas are specialized to recognize places [66], faces [67] and objects [63]. The putamen projects to the cortical motor areas, including the SMA, via the globus pallidus [68]. In Parkinson’s Disease, the dopamine uptake is reduced in the striatum and particularly in the putamen [69]. The putamen has shown decreased regional homogeneity in patients with Parkinson’s Disease as compared to controls in the resting state [25] and meta-analysis on functional neuroimaging data show that the putamen is indeed the nucleus whose decreased function consistently correlates with motor deficits in Parkinson’s Disease OFF patients [62]. The putamen is involved in the planning of self-initiated or self-paced movements [70] and the anterior striatum is involved, together with pre-SMA, in the preparation and updating of plans of future actions that are under voluntary control [71]. On this respect it is interesting that functional modulation after effective AMPS showed increased connectivity of the striatum with areas that are part of the ventral visual processing stream but not with brainstem-cerebellum or executive motor fronto-parietal areas as expected [28, 59]. The nucleus striatum also showed a stable increase of functional connectivity after AMPS with the right temporal lobe, an area that is interchangeably named to indicate what is functionally known as anterior temporal lobe [72]. The anterior temporal lobe’s function has been explored in many studies from which one of the most recognized models is the so called “semantic hub” theory [73]. On this basis the anterior temporal lobe could act as a multimodal semantic hub where information from different sensory (visual, somatosensory and auditory) modalities converges [73]. In this context, the lateralization of the increased coherence consistently observed between the nucleus striatum and the right anterior temporal lobe rather than the left one would fit well with the emerging evidence that in conceptual knowledge the right anterior temporal lobe is specialized for visual recognition whereas the left one is specialized for the process of lexical access [74, 75]. Previous studies have shown that: i. there is decreased fMRI activation of the visual pathway in Parkinson’s Disease in the absence of clinically relevant visual symptoms [76]; ii. patients with Parkinson’s Disease rely on visual cues to control locomotion [77, 78]; iii. Parkinson’s Disease patients with freezing of gait show reduced resting state connectivity at the right occipito-temporal gyrus in respect to Parkinson’s Disease patients without freezing of gait [9]. Thus, our results support the idea that visuo-spatial integration significantly impact on motor execution in Parkinson’s Disease patients and its function may be modulated by peripheral stimulation. Moreover, the lack of changes in the cortico-basal ganglia motor loop connectivity suggests that functional pathways different than those dependent on the dopaminergic system [60, 62, 79] are modulated after AMPS.

### Cerebellum Functional Connectivity

The functional connectivity of the cerebellum increased after effective AMPS. The effect involved again the right occipital fusiform gyrus and the right lateral occipital cortex. The connectivity between primary motor, pre-motor, supplementary motor cortices and cerebellum has shown to be disrupted in Parkinson’s Disease patients compared to controls during performance of self-initiated movements. In that paper, Wu et al. also showed that, while striatum-cortical and striatum-cerebellar connections are weakened in Parkinson’s Disease patients, cortico-cerebellar connections are strengthened and may compensate for nigro-striatal dysfunction [58]. We observed modulation of functional connectivity between the cerebellum and areas of the visual ventral processing system, thus suggesting that peripheral stimulation may have a facilitating effect on the function of compensatory areas. Moreover, the mixed model showed an intra-regional cerebellar increased connectivity with the right crus I that was confirmed even when left and right cerebellum hemispheres were separately tested as seed regions of interest. Clusters of significantly increased connectivity after stimulation were located at the level of the paravermal lobules, functionally known as part of the spino-cerebellum. The spino-cerebellum receives extensive sensory inputs from the dorsal columns of the spinal cord and sends efferent fibers to the cerebellar deep nuclei to integrate sensory-motor information and allows anticipation of body position during movement in a feed-forward manner [80]. Moreover, according to the somatotopic organization of the spino-cerebellum, the paravermal intermediate areas, as we observed, are involved in the control of legs and arms movements.

The cerebellum physiologically enters into action when preparing or executing movements [81], and is also involved in generating accurate timing of self-paced movements [82]. fMRI studies have consistently showed increased activation in the cerebellum of patients with Parkinson’s Disease OFF medication during the performance of motor tasks [83] and in the resting state in patients with akinesia/rigidity [25]. Analysis of connectivity based on the graph theory has shown increased connectivity degrees in the left cerebellum, together with the left premotor cortex and left parietal cortex in patients OFF medication, partially normalized by administration of levodopa [84].

Parkinson’s Disease patients have shown recruitment of cerebellum activity to increase movement velocity as a mechanism of compensation for basal ganglia dysfunction [85–86]. However, the idea of a compensatory function of the cerebellum is still under debate and physiopathological mechanisms such as the inability to inhibit activity secondary to abnormal basal ganglia function cannot be ruled out [87–88]. Nevertheless, our observation of increased connectivity of the cerebellum supports the hypothesis of a cerebellar compensatory effect in Parkinson’s Disease and that AMPS may facilitate these neural circuits.

### Effective versus Sham Plantar Mechanical Stimulation

The sham stimulation did not yield significant changes of functional connectivity with any of the chosen seed ROIs. However, to correct for any sub-threshold effects induced by sham stimulation, we performed direct comparisons of functional connectivity in the effective and sham conditions using a repeated-measures mixed-effects model. We found a stronger level of connectivity with the nuclei striati and the cerebellum, but not with the sensory motor cortex, the supplementary motor area and basal ganglia. Particularly, the functional connectivity of the nuclei striati was significantly stronger with the right lateral occipital cortex and with the right temporal pole while the cerebellum showed stronger connectivity intra-regionally in the right crus I and again with the right lateral occipital cortex. These latter findings reflect the net result of connectivity changes after effective stimulation. This analysis confirmed intra-regional functional coupling in the paravermal cerebellum and between the cerebellum and the ventral visual processing, especially the lateral occipital complex.

Moreover, the nuclei striati showed consistent functional coupling with the same areas discussed above and, following AMPS, stronger connectivity was found between the nuclei striati and the cuneal cortex, which is also known to be involved in visual processing.

### Interpretational Issues

The main limitation of the present pilot study is the small number of subjects included, further restricted for the fMRI analyses. However, the results of first-level (datasets number = 14) and second-level (datasets number = 28) analyses were consistent and were confirmed with and without inclusion of 6 head motion parameters in the analysis. In the current study we explored functional connectivity with a seed-ROI based approach using the brain regions known to be the most involved in Parkinson Disease physiopathology as seeds. However, a data driven approach is needed to obtain information on functional brain networks without a priori hypotheses. Moreover, false negative results may have occurred in this study and trials conducted on large groups of patients are necessary to fully understand the complex effects of peripheral stimulation on brain functional connectivity at rest.

Despite our seed ROIs were bilateral, significant differences of functional connectivity showed lateralization to the left side for the cerebellum vermis and the primary sensory motor cortex and to the right side for the nucleus striatum and cerebellum hemispheres. In the case of the visual ventral pathway, the lateralization may be explained by the well-known right hemisphere predominance of visuospatial skills in the human brain [89]. As it regards the right lateralization of the effect in the cerebellum, we currently have not an unequivocal explanation: 1. the right side of the cerebellum is strongly connected with the contralateral left sensory-motor pathway and its function could be preferentially modulated by the plantar stimulation in this group of right-handed patients; 2. lack of changes on the left side of the cerebellum could be a false negative result due to the small size of the sample; 3. other mechanisms of cerebellar functional asymmetry may exist [90].

In the future, our investigation will be aimed to increase the sample size of Parkinson’s Disease patients undergoing AMPS and to explore the effects of the peripheral stimulation on healthy control subjects.

## Conclusions

In conclusion, our results showed a consistent effect of AMPS on increasing resting state functional connectivity (RSFC) of brain regions involved in visuo-spatial integration and processing, in sensory-motor integration and anticipation of body position during movements. These effects are associated with improvement of the ability to initiate voluntary movements of the lower limbs in Parkinson’s Disease patients OFF medication, thus suggesting that AMPS may have a role in rehabilitative protocols by facilitating brain compensatory pathways to manage symptoms such as akinesia.

## Supporting Information

S1 TREND Checklist TREND Checklist. (PDF)

S1 Clinical Trials Pilot Registration of the study from the ClinicalTrials.gov
(PDF)

S1 Database Clinical data. (XLS)

S1 Protocol Study protocol approved by theEthical Committee (Italian). (PDF)

S2 Protocol Study protocol approved by the Ethical Committee (English). (PDF)

S1 Results Additional Results. (DOC)
