# Supplementary material for: Acute Modulation of Brain Connectivity in Parkinson Disease after Automatic Mechanical Peripheral Stimulation: A Pilot Study
Source: PLoS One. 2015 Oct 15;10(10):e0137977. doi: 10.1371/journal.pone.0137977 (PMC4607499; doi:10.1371/journal.pone.0137977)
Supplement: S1 Clinical Trials Pilot — ClinicalTrials.gov (PDF) [file pone.0137977.s002.pdf]

ClinicalTrials.gov PRS
Protocol Registration and Results System

ClinicalTrials.gov Protocol and Results Registration System (PRS) Receipt
Release Date: 03/16/2015

Foot Mechanical Stimulation for Treatment of Gait and Gait Related Disorders in Parkinson's Disease and Progressive Supranuclear Palsy. (GONDOLAPILOTA)

This study is currently recruiting participants.
Verified by Patrizio Sale , MD, IRCCS San Raffaele, March 2015

Sponsor:	IRCCS San Raffaele
Collaborators:	
Information provided by (Responsible Party):	Patrizio Sale , MD, IRCCS San Raffaele
ClinicalTrials.gov Identifier:	NCT01815281

Purpose

The purpose of this research study is to evaluate safety and effectiveness of Foot Mechanical stimulation to improving Gait and Gait Related Disorders in Parkinson Disease and Progressive Supranuclear Palsy both stable and with motor fluctuation.

Condition	Intervention	Phase
Idiopathic Parkinson's Disease<br>Progressive Supranuclear Palsy	Device: Foot Mechanical Stimulation (GONDOLA)	N/A

Study Type: Interventional
Study Design: Treatment, Crossover Assignment, Double Blind (Subject, Caregiver, Investigator, Outcomes Assessor), Randomized, Safety/Efficacy Study
Official Title: Foot Mechanical Stimulation for Treatment of Gait and Gait Related Disorders in Parkinson's Disease

Further study details as provided by Patrizio Sale , MD, IRCCS San Raffaele:

Primary Outcome Measure:

- Timed Up and Go. [Time Frame: Change from Baseline in Timed Up and Go test at 1 month follow up.] [Designated as safety issue: Yes]

  Time Up and Go test will be collected in OFF state 4 hour after oral assumption of levodopa at baseline (inclusion) (T0), before (T1-T3-T5-T7-T9-T11) and after all stimulation (T2-T4-T6-T8-T10) and endpoint (after 6 stimulation) (T12) at the follow-up examination after 1 months from the treatments conclusion (T13).

Secondary Outcome Measures:

- 6 minuts walking test. [Time Frame: Change from Baseline in gait speed at 1 month follow up] [Designated as safety issue: Yes]

6 minuts walking test will be collected in OFF state 4 hour after oral assumption of levodopa at baseline (inclusion) (T0), before (T1-T3-T5-T7-T9-T11) and after all stimulation (T2-T4-T6-T8-T10) and endpoint (after 6 stimulation) (T12) at the follow-up examination after 1 months from the treatments conclusion (T13).

- Gait Parameters [Time Frame: Change from Baseline in Gait Parameters at 1 month follow up] [Designated as safety issue: Yes]

  Gait Analysis will be collected in OFF state 4 hour after oral assumption of levodopa at baseline (inclusion) (T0), before (T1-T3-T5-T7-T9-T11) and after all stimulation (T2-T4-T6-T8-T10) and endpoint (after 6 stimulation) (T12) at the follow-up examination after 1 months from the treatments conclusion (T13).
- FREEZING OF GAIT QUESTIONNAIRES [Time Frame: Change from Baseline in FREEZING OF GAIT QUESTIONNAIRES at 1 month follow up.] [Designated as safety issue: Yes]
- THE PARKINSON'S DISEASE QUESTIONNAIRE (PDQ-39) [Time Frame: Change from Baseline in PDQ-39 at 1 month follow up] [Designated as safety issue: Yes]

Other Pre-specified Outcome Measures:

- UNIFIED PARKINSON'S DISEASE RATING SCALE (UPDRS). [Time Frame: Change from Baseline in UPDRS scores at 1 month follow up] [Designated as safety issue: Yes]
- PROGRESSIVE SUPRANUCLEAR PALSY RATING SCALE (PSP RATING SCALES) [Time Frame: Change from Baseline in PSP RATING SCALES scores at 1 month follow up] [Designated as safety issue: Yes]
- Functional Ambulation classification (FAC) [Time Frame: Change from Baseline in FAC scores at 1 month follow up] [Designated as safety issue: Yes]
- Walking handicap Scale (WHS) [Time Frame: Change from Baseline in WHS scores at 1 month follow up] [Designated as safety issue: Yes]
- BOLD signal response to gondola treatment [Time Frame: Change from Baseline in UPDRS scores at 1 month follow up] [Designated as safety issue: No]

  BOLD signal will be collected in OFF state 4 hour after oral assumption of levodopa at baseline (inclusion) (T0), before (T1-T3-T5-T7-T9-T11) and after all stimulation (T2-T4-T6-T8-T10) and endpoint (after 6 stimulation) (T12) at the follow-up examination after 1 months from the treatments conclusion (T13).

Study Start Date: July 2013
Primary Completion Date: November 2013
Estimated Study Completion Date: April 2015

Arms	Assigned Interventions
Experimental: Foot Mechanical Stimulation<br>The FMS stimulation will be given to all participants using GONDOLA equipment (Ecker Technologies Sagl, Switzerland).	Device: Foot Mechanical Stimulation (GONDOLA)<br>Other Names:<br>GONDOLA equipment (Ecker Technologies Sagl, Switzerland).
Sham Comparator: Footh Mechanical Stimulation<br>The sham stimulation will be given to all participants using GONDOLA equipment (Ecker Technologies Sagl, Switzerland).	Device: Foot Mechanical Stimulation (GONDOLA)<br>Other Names:<br>GONDOLA equipment (Ecker Technologies Sagl, Switzerland).

Eligibility

Ages Eligible for Study: 18 Years to 90 Years
Genders Eligible for Study: Both
Accepts Healthy Volunteers: Yes

Criteria

Inclusion Criteria:

- Diagnosis of idiopathic PD or PSP by UK Brain Bank criteria,
- Able to walk 25 feet unassisted or with minimal assistance;
- On stable doses of Parkinson's medications for at least 2 weeks prior to study onset;
- Endurance sufficient to stand at least 20 minutes unassisted per patient report.

Exclusion Criteria:

- Other significant neurological or orthopedic problems.

Contacts and Locations

Locations

Italy

San Raffaele Cassino **Recruiting**

Cassino (FR), Italy
Contact: Maria Francesca De Pandis, MD maria.depandis@sanraffaele.it
Principal Investigator: Maria Francesca De Pandis, MD
Sub-Investigator: Patrizio Sale, MD

IRCCS San Raffaele Pisana **Recruiting**

Rome, Italy, 00163
Contact: Patrizio Sale, MD patrizio.sale@gmail.com
Principal Investigator: Fabrizio Stocchi, MD
Sub-Investigator: Patrizio Sale, MD
Sub-Investigator: Laura Vacca, MD

University Campus Biomedico of Rome **Not yet recruiting**

Rome, Italy, 00128
Contact: Carlo Cosimo Quattrocchi, MD c.quattrocchi@unicampus.it
Principal Investigator: Carlo Cosimo Quattrocchi, MD

Investigators

Study Chair:	Fabrizio Stocchi, MD	IRCCS San Raffaele Pisana Rome Italy
Study Director:	Patrizio Sale, MD	IRCCS San Raffaele Pisana Rome Italy
Principal Investigator:	Fabrizio Stocchi, MD	IRCCS San Raffaele Pisana Rome Italy

More Information

Responsible Party:	Patrizio Sale , MD, MD, IRCCS San Raffaele
Study ID Numbers:	GONDOLAPILOTA
Health Authority:	Italy: Ministry of Health
