# Supplementary material for: Acute Modulation of Brain Connectivity in Parkinson Disease after Automatic Mechanical Peripheral Stimulation: A Pilot Study
Source: PLoS One. 2015 Oct 15;10(10):e0137977. doi: 10.1371/journal.pone.0137977 (PMC4607499; doi:10.1371/journal.pone.0137977)
Supplement: S1 Protocol — (PDF) [file pone.0137977.s004.pdf]

IRCCS San Raffaele
Pisana

Presidio Ospedaliero: via della Pisana, 235 | 00163 Roma | Centralino 06 660581 | irccs@sanraffaele.it
Poliambulatori Specialistici: via della Pisana, 235 | 00163 Roma | Via Giolitti, 16 | 00185 Roma | C.U.P. 89 22 89*
Centro di Riabilitazione: via della Pisana, 216 | 00163 Roma | Tel. 06 66058321
Laboratorio analisi con settori specializzati: via dei Bonacolsi, 81 | 00163 Roma | Tel. 06 66130400
Centro di Formazione: via di Val Cannuta, 247 | 00166 Roma | Tel. 06 5225 2454
www.sanraffaele.it

PROTOCOLLO DI STUDIO

Roma, 20 Maggio 2013

Ricerca con dispositivo medico dal titolo: **Studio pilota cross-over sull'utilizzo della "Gondola" per la riabilitazione motoria di soggetti affetti da malattia di Parkinson e da Paralisi Sopranucleare Progressiva - codice "GONDOLA PILOTA**

Principal Investigator: Prof. Fabrizio Stocchi.

BACKGROUND, AIMS AND IMPACT

Background:

Gait dysfunction is a cardinal feature of Parkinsons disease (PD) and Progressive supranuclear palsy (PSP).
The progressive loss of the neurotransmitter dopamine leads to an imbalance in the basal ganglia and PD patients experience difficulties with motor skills, bradykinesia, rigidity, tremor and postural instability. Sensory, emotional, and perceptual signs also are observed in some individuals.
Footsteps become asymmetrical and underscaled in size and speed (hypokinesia), difficult to activate (akinesia), and difficult to terminate and show progressive diminution as the locomotor sequence progresses (Morris 2000).
Such impairments of body structure and body function are associated with limitations in activities of daily living (ADL), such as walking, moving from a lying to sitting to standing position (Muslimovic 2008). Functional activities become compromised, even though the ability to perform simple movements is retained.
Impairments and activity limitations following PD and PSP restrict the persons ability to participate in societal roles related to work, family life, education, civic life, and leisure. Although levodopa and other PD medications are initially very effective in reducing the severity of movement disorders, some gait disorders persist despite optimal medication (Jankovic 2000, Motto 2003). In particular, the freezing phenomenon, a gait disorder in which patients are unable to initiate or continue locomotion, is very difficult to treat. The pharmacological treatment is usually disappointing: whereas patients with freezing in off states can gain benefit from an increase in levodopa dosage, this was not observed in patients with freezing in on states.
The effectiveness of non-pharmacological options such as exercises has been demonstrated (Goodwin 2008); in particular an example for patient tailored exercises is physiotherapy (Ashburn 2004; Comella 1994; de Goede 2001).
Several systematic reviews and clinical studies have shown that physical therapy can contribute to minimize the disabling effects of motor and sensory impairments in order to enhance participation in societal roles and quality of life. The goal of physiotherapists is to enable PD patients to maintain their maximum level of mobility, activity, and independence.

PIS 1200000039/001

Gestito da San Raffaele S.p.A. | Via di Val Cannuta, 247 - 00166 Roma | PEC: sanraffaele.fiscale@legalmail.it
PI 08253151008 | REA Roma 1083742 | C.F. e Iscriz. Registro Imprese n. 08253151008 | Capitale Sociale 1.000.000,00 euro i.v.

* Numero a tariffazione specifica di pubblica utilità in ambito sanitario. Il costo della telefonata, comprensivo d'IVA, da rete fissa è di 12 centesimi di Euro al minuto, da cellulari TIM 48 cent di Euro al minuto più scatto alla risposta di 16 cent, VODAFONE 48 cent di Euro al minuto più scatto alla risposta di 16 cent, WIND 48 cent di Euro al minuto più scatto alla risposta di 15 cent, H3G 48 cent di Euro al minuto più scatto alla risposta di 16 cent. Il servizio ha una durata massima da rete fissa di 125 minuti e per un costo massimo di 15 euro. Il servizio è destinato ai maggiorenni ed è erogato dal San Raffaele spa.

IRCCS San Raffaele
Pisana

Presidio Ospedaliero: via della Pisana, 235 | 00163 Roma | Centralino 06 660581 | irccs@sanraffaele.it
Poliambulatori Specialistici: via della Pisana, 235 | 00163 Roma | Via Giolitti, 16 | 00185 Roma | C.U.P. 89 22 89*
Centro di Riabilitazione: via della Pisana, 216 | 00163 Roma | Tel. 06 66058321
Laboratorio analisi con settori specializzati: via dei Bonacolsi, 81 | 00163 Roma | Tel. 06 66130400
Centro di Formazione: via di Val Cannuta, 247 | 00166 Roma | Tel. 06 5225 2454
www.sanraffaele.it

The use of electromechanical devices (a supplement to conventional therapies) in the last years has also been used with PD patients (Miyai 2002, Pohl 2003).

The purpose of this research study is to evaluate safety and effectiveness of Foot Mechanical stimulation to improving Gait and Gait Related Disorders in patients with Parkinson's disease (PD) and Progressive supranuclear palsy (PSP).
Gondola® is a portable device that runs on batteries and fits like a shoe. It was designed for the stimulation of two areas of both feet (first toe and metatarsal) through mechanical impulses set up for pressure, duration and sequence (Foot Mechanical Stimulation).

The use of quantitative movement analysis allows an objective multifactorial evaluation of the functional limitation related to the pathology as well as a quantitative outcome evaluation after a specific treatment.

In particular the use of the movement analysis lab in subjects with PD and PSP treated with GONDOLA treatments, can lead to better understanding of the following:

1. the motor impairments of the subjects with PD or PSP during the execution of specific movements like gait, posture;
2. the specific district requiring a specific treatment;
3. the selection of kinematic pattern to be imposed by the GONDOLA on the treated subjects during the treatment;
4. the multifactorial quantitative analysis of outcomes following the treatment and to compare the rehabilitative techniques with the support of statistical data analysis.

Aims

The specific aims of this project are:

1. to verify whether the lower limb treatment with GONDOLA is effective in the reduction of motor impairment in PD or PSP patients, and to improve the quality of the gait and the endurance;
2. to analyze possible improvements in terms of physiological biomechanical gait through analysis of kinematics and kinetics;
3. to analyze possible improvements in terms of reduction of instable posture and movements, which can represent a reduction of the risk of falls typical of these subjects;
4. to investigate the stability of the effects of GONDOLA treatment in terms of Quality of Life (QoL).

A first goal of this project is to investigate the differences in improvement of the quality and safety of the gait (motor performance and functional recovery) through kinematic/kinetic (Change in Step Length, Change in Gait Velocity and Change in Stride Time Variability, 3D joints kinematics, ground reaction forces, joint kinetics,) and traditional clinical scales in Parkinson's and PSP patients.

PIS 12000000839/001

Gestito da San Raffaele S.p.A. | Via di Val Cannuta, 247 - 00166 Roma | PEC: sanraffaele.fiscale@legalmail.it
PI 08253151008 | REA Roma 1083742 | C.F. e Iscriz. Registro Imprese n. 08253151008 | Capitale Sociale 1.000.000,00 euro i.v.

* [illegible]

IRCCS San Raffaele
Pisana

Presidio Ospedaliero: via della Pisana, 235 | 00163 Roma | Centralino 06 660581 | irccs@sanraffaele.it
Poliambulatori Specialistici: via della Pisana, 235 | 00163 Roma | Via Giolitti, 16 | 00185 Roma | C.U.P. 89 22 89*
Centro di Riabilitazione: via della Pisana, 216 | 00163 Roma | Tel. 06 66058321
Laboratorio analisi con settori specializzati: via dei Bonacolsi, 81 | 00163 Roma | Tel. 06 66130400
Centro di Formazione: via di Val Cannuta, 247 | 00166 Roma | Tel. 06 5225 2454
www.sanraffaele.it

The second goals is aimed at identifying possible advantages in the QoL of patients undergoing such a kind of rehabilitation treatment and at investigating novel methods enabling lower limb functional recovery, leading to wide potential for regaining personal independence.

Main Expected Results and Impact
Clinical assessment scales represent the most common outcome measures in rehabilitation so far; they provide merely quantitative information on the patients motor performance, but are unable to provide qualitative information, which could be useful in differentiating the mechanisms underlying motor recovery.

Primary Outcome Measures: The primary outcome measures will be the change in the gait composite score (UPDRS, PSP rating scale, Timed up and go and GAIT analysis) between the FMS and Placebo (Sham) groups.

Secondary Outcome Measures:
Secondary outcome measures include:
- FREEZING OF GAIT QUESTIONNAIRES
- THE SIX-MINUTE WALK TEST
- TEN METER WALK TEST (TMWT)
- THE PARKINSON'S DISEASE QUESTIONNAIRE (PDQ-39)
- EQ-5D
- HAMILTON RATING SCALE FOR DEPRESSION
- NMS scale (Non Motor Symptoms scale)
- MoCA (Montreal Cognitive Assessment)
- CLINICAL GLOBAL IMPRESSION OF CHANGE FOR INVESTIGATOR AND PATIENT/CAREGIVER (CGI-I E CGI-P/CG)
- CHANGING IN FUNCTIONAL NMR

Methodology

Study Design
The proposed project, through a randomized controlled observer-blind cross-over trial aimed at evaluating the effectiveness of GONDOLA therapy versus SHAM GONDOLA therapy in PD or PSP subjects. 30 in/outpatients (20 patients with a PD and 10 patients PSP) will be recruited. We will randomize the patients on 2 groups GONDOLA therapy versus SHAM GONDOLA. At the end of the first 6 stimulation (GONDOLA OR SHAM), subject will undergo a washout period of 3 weeks followed by a second cycle of treatments (SHAM OR GONDOLA). At the end of the trial all patients will have performed both kind of treatments. A Gait Analysis will be perform in at least 50% of patients with PD and all PSP at the end of each treatment cycle. Functional NMR was performed in a subgroup of patients .

Bibliographic Data
1. Sale P, Semprini R, Foti C, Fini M, Franceschini M. Gait impairment in neurological disorders: a new technological approach. Funct Neurol. 2009 Oct-Dec;24(4):179-83.

PRS 120[illegible]/001

Gestito da San Raffaele S.p.A. | Via di Val Cannuta, 247 - 00166 Roma | PEC: sanraffaele.fiscale@legalmail.it
PI 08253151008 | REA Roma 1083742 | C.F. e Iscriz. Registro Imprese n. 08253151008 | Capitale Sociale 1.000.000,00 euro i.v.

* Numero a tariffazione specifica di pubblica utilità in ambito sanitario. Il costo della telefonata, comprensivo d'IVA, da rete fissa è di 12 centesimi di Euro al minuto, da cellulari TIM 40 cent di Euro al minuto più scatto alla risposta di 15 cent, VODAFONE 48 cent di Euro al minuto più scatto alla risposta di 15 cent, WIND 48 cent di Euro al minuto più scatto alla risposta di 15 cent, H3G 48 cent di Euro al minuto più scatto alla risposta di 16 cent. Il servizio ha una durata massima da rete fissa di 120 minuti e per un costo massimo di 15 euro. Il servizio è destinato ai maggiorenni ed è erogato dal San Raffaele spa.

2. Winchester P, Querry R: Robotic orthoses for body weight-supported treadmill training. Physical medicine and rehabilitation clinics of North America 2006, 17:159-172.;
3. Volpe BT, Krebs HI, Hogan N. Is robot-aided sensorimotor training in stroke rehabilitation a realistic option? Curr Opin Neurol. 2001;14: 745752.
4. Davis R B, Ounpuu S, Tyburski D J &Gage JR. A gait analysis data collection and reduction technique. Human Movement Science 1991; 10 (5):
575-587 Morris ME, Huxham F, Mc Ginley J, Dodd K, Iansek R. the biomechanics and motor control of gait in PD. Clinical Biomechanics 2001; 16: 459-470
5. Nieuwboer A, Dom R, De WW, Desloovere K, Fieuws S, Broens-Kaucsik E. Abnormalities of the spatiotemporal characteristics of gait at the onset of freezing in Parkinson's disease. Movement Disorders 2001; 16: 10661075.
6. Morris M, Iansek R, McGinley J, Matyas T, Huxham F. Three- dimensional gait biomechanics in Parkinsons disease: evidence for centrally mediated amplitude regulation disorder. Movement Disorders 2005; 20 (1): 4050
7. Hausdorff JM. Gait dynamics, fractals and falls: finding meaning in the stride-to-stride fluctuations of human walking. Human Movement Science. 2007; 26 (4): 555589
8. Morris M. Movement disorders in people with Parkinson disease: a model for physical therapy. Physical Therapy 2000;80:578-97.
9. Muslimovic D, Post B, Speelman JD, Schmand B, de Haan RJ. Determinants of disability and quality of life in mild to moderate Parkinson disease. Neurology 2008; Vol. 70, issue 23:22417.
10. Jankovic J. Complications and limitations of drug therapy for Parkinsons disease. Neurology 2000; Vol. 55, issue Suppl 6 (12):S26.
11. Goodwin VA, Richards SH, Taylor RS, Taylor AH, Campbell JL. The effectiveness of exercise interventions for people with Parkinsons disease: a systematic review and meta-analysis. Movement Disorders 2008; Vol. 23, issue 5: 63140.
12. Ashburn A, Jones D, Plant R, Lovegreen B, Kinnear E, Handford F, et al.Physiotherapy for people with Parkinsons disease in the UK: an exploration of practice. International Journal of Therapeutics and Rehabilitation 2004; Vol. 11, issue 4:167.
13. Pohl M, Rockstroh G, Ruckriem S, Mrass G, Mehrholz J. Immediate effects of speed-dependent treadmill training on gait parameters in early Parkinsons disease. Archives of Physical Medicine and Rehabilitation 2003;84:17606.
14. Mehrholz J, Friis R, Kugler J, Twork S, Storch A, Pohl M. Treadmill training for patients with Parkinsons disease. Cochrane Database of Systematic Reviews 2010, Issue 1. Art. No.: CD007830. DOI: 10.1002/14651858.CD007830.pub2.
15. Lo AC, Chang VC, Gianfrancesco MA, Friedman JH, Patterson TS, Benedicto DF. Reduction of freezing of gait in Parkinson's disease by repetitive robot-assisted treadmill training: a pilot study. J Neuroeng Rehabil. 2010 Oct 14;7:51.

Work Methodology

Inclusion criteria:
Diagnosis of idiopathic PD by UK Brain Bank criteria and PSP according to NINDS-SPSP criteria, without other significant neurological or orthopedic problems; between the ages of 18-80; able to

Gestito da San Raffaele S.p.A. | Via di Val Cannuta, 247 - 00166 Roma | PEC: sanraffaele.fiscale@legalmail.it
PI 08253151008 | REA Roma 1083742 | C.F. e Iscriz. Registro Imprese n. 08253151008 | Capitale Sociale 1.000.000,00 euro i.v.
[illegible]

IRCCS San Raffaele
Pisana

Presidio Ospedaliero: via della Pisana, 235 | 00163 Roma | Centralino 06 660581 | irccs@sanraffaele.it
Poliambulatori Specialistici: via della Pisana, 235 | 00163 Roma | Via Giolitti, 16 | 00185 Roma | C.U.P. 89 22 89*
Centro di Riabilitazione: via della Pisana, 216 | 00163 Roma | Tel. 06 66058321
Laboratorio analisi con settori specializzati: via dei Bonacolsi, 81 | 00163 Roma | Tel. 06 66130400
Centro di Formazione: via di Val Cannuta, 247 | 00166 Roma | Tel. 06 5225 2454
www.sanraffaele.it

walk 25 feet unassisted or with minimal assistance; on stable doses of Parkinson's medications for at least 2 weeks prior to study onset.

Power sample
Based on a preliminary power analysis (p=0.005) using archive spatiotemporal and kinematic data previously recorded in a small group of patients, we will recruit and randomize in 2 groups 30 in/outpatients (20 patients with a PD and 10 PSP patients).

Clinical outcome measures
Primary end-point: improvement in the UPDRS part II and III.
All outcome motor assessments will be collected in off phase at baseline (T0-T2) and endpoint (T1-T3), and at the follow-up examination after 4 weeks after treatments conclusion (T4).
They will include:
Unified Parkinson's Disease Rating Scale.
Hoehn and Yahr Stage
PSP Rating Scale and Staging System
The Freezing of Gait-Questionnaire (FOG-Q).
Time Up and Go test
6 Minute Walk Test.
10 meter walk test.
Multifactorial 3D gait and posture analysis.
All cognitive, depressive and quality of life assessments will be performed in on state:
Hamilton Rating Scale for Depression
Non Motor Symptoms Scale
MoCA (Montreal Cognitive assessment)
Clinical Global Impression of change for investigator and patient/caregiver (CGI-I e CGI-p/cg)

Random group allocation
The random allocation to 2 groups of treatment will be concealed and based upon a custom computerized system exploiting software purposely realized for this research.

Treatment
Trained professionals, not directly involved in the research treatment and blind to patients group allocation, will perform clinical assessments.
All subjects will undergo inpatient rehabilitation consisting of a two cross-over treatment cycles using the GONDOLA device or the SHAM GONDOLA.

Each cycle consists of 6 sessions for the lower limbs, 2 days a week for 3 weeks.

Quantitative biomechanical data recording (kinematics and kinetics):
Biomechanical data will be recorded at the beginning (T0-T2), at the end of the treatment period weeks (T1-T3) in at least 50% of patients with PD and all PSP.
A follow-up clinical evaluation will be performed 4 weeks after last cycle (T4).

Gestito da San Raffaele S.p.A. | Via di Val Cannuta, 247 - 00166 Roma | PEC: sanraffaele.fiscale@legalmail.it
PI 08253151008 | REA Roma 1083742 | C.F. e Iscriz. Registro Imprese n. 08253151008 | Capitale Sociale 1.000.000,00 euro i.v.

* Numero a tariffazione specifica di pubblica utilità in ambito sanitario. Il costo della telefonata, comprensivo d'IVA, da rete fissa è di 12 centesimi di Euro al minuto, da cellulari TIM 49 cent di Euro al minuto più scatto alla risposta di 16 cent, VODAFONE 49 cent di Euro al minuto più scatto alla risposta di 15 cent, WIND 49 cent di Euro al minuto più scatto alla risposta di 15 cent, H3G 49 cent di Euro al minuto più scatto alla risposta di 16 cent. Il servizio ha una durata massima da rete fissa di 125 minuti e per un costo massimo di 15 euro. Il servizio è destinato ai maggiorenni ed è erogato dal San Raffaele spa.

Correlation between Biomechanical Parameters and Clinical Scores
In order to evaluate the correlation between biomechanical parameters and clinical scale scores, statistical correlations will be computed between changes in each metric and corresponding changes in clinical scales (UPDRS, TUG, 6MWT 10mWT) at T0, T1 and T2, T3, T4.
Pearsons product-moment coefficient of correlation will be used for parametric data, and Spearmans rank correlation coefficient will be used for non-parametric data.

Workplan:
WP1 Project coordination and management (Month 1 to 18)
WP2 Definition of clinical protocol and patient recruitment (Month 1 to 10)
WP3Multicentre Clinical Trials (Month 1 to 10)
WP4 Development of methods and tools for clinical data analysis (Month 14 to 16)
WP5 Clinical data analysis and interpretation (Month 14 to 18)

Milestones
M1 (month1): Approval of research proposal (Ethics Committee) and preliminary activities.
M2 (month 3): Electronic sheet for data collection
M3 (month 4): Demonstration of the integrated set up by a pilot study (Interim analysis)
M4 (month 10): Completion of the RCT.
M5 (month 14): Description of methods and tools for data analysis
M6 (month 13): Completion of the follow-up study.
M7 (month 14): Data analysis and interpretation.

Equipment: IRCCS San Raffaele Pisana Rome and San Raffaele Cassino (Italy) are equipped with the following instruments:

Equipment for the quantitative analysis evaluation. Gait analysis and Posture evaluation.
IRCCS San Raffaele Pisana Rome Italy is equipped with the following instruments:
Optoelectronic system: this device measures the three-dimensional coordinates of markers that are placed at reference points of the subjects body.
This enables to calculate trajectories, angular ranges, velocities, accelerations and therefore to define kinematics of the single corporeal segment on which the markers are positioned.
The IRCCS San Raffaele Pisana Rome and San Raffaele Cassino, Italy are equipped with a 12-camera ELITE 2002 system (BTS, Milan, Italy) for total body movements, and a 6-camera SMART system (BTS, Italy) for fine movements.

Force platforms: system that measures the ground reaction forces. By knowing the ground reaction forces, together with the kinematics (that is acquired by the optoelectronic system) it is therefore possible to compute the moments and powers at the different joints. Two force platforms (Kistler, CH) are available.

Experimental set-up for **Motion analysis**

IRCCS San Raffaele
Pisana

Presidio Ospedaliero: via della Pisana, 235 | 00163 Roma | Centralino 06 660581 | irccs@sanraffaele.it
Poliambulatori Specialistici: via della Pisana, 235 | 00163 Roma | Via Giolitti, 16 | 00185 Roma | C.U.P. 89 22 89*
Centro di Riabilitazione: via della Pisana, 216 | 00163 Roma | Tel. 06 66058321
Laboratorio analisi con settori specializzati: via dei Bonacolsi, 81 | 00163 Roma | Tel. 06 66130400
Centro di Formazione: via di Val Cannuta, 247 | 00166 Roma | Tel. 06 5225 2454
www.sanraffaele.it

The acquisition will be performed according standard and international protocol in terms of marker position. As concerns Gait Analysis and posture analysis acquisition the marker set will be according to Davis et al. (1991).

Gait Analysis and posture analysis:
After collecting the anthropometric measures (height, weight, tibial length, distance between femoral condyles or diameter of the knee, distance between malleoli or diameter of the ankle, distance between anterior iliac spines and thickness of the pelvis), passive markers will be placed at special reference points (Davis, 1991) on the subjects skin.

Thereafter, two acquisitions sets will be obtained:
Gait Analysis: the patient will be asked to walk barefoot at his self-selected and convenient speed along a 10-metre walkway. Seven trials will be recorded during analysis in order to reduce variability and improve data consistency.

Posture Analysis: each patient will be standing on a force platform for 60 seconds being their feet positioned at an angle of 30° with respect to the antero-posterior direction. Patient will be asked to stand motionless while keep their eyes opened (OE) and focus on a 6 cm black circle positioned at the individual line of vision, at 1.5 m distance. Then the subject will be asked to repeat the same task while closing eyes (CE). Two trials will be acquired both with OE and CE. Between trials, the subject will be allowed to sit for 2 minutes for full recovery.

Principal Investigator: Prof. Fabrizio Stocchi.

Signature:

Gestito da San Raffaele S.p.A. | Via di Val Cannuta, 247 - 00166 Roma | PEC: sanraffaele.fiscale@legalmail.it
PI 08253151008 | REA Roma 1083742 | C.F. e Iscriz. Registro Imprese n. 08253151008 | Capitale Sociale 1.000.000,00 euro i.v.

* Numero a tariffazione specifica di pubblica utilità in ambito sanitario. Il costo delle telefonate, comprensivo d'IVA, da rete fissa è di 12 centesimi di Euro al minuto, da cellulari TIM 49 cent di Euro al minuto più scatto alla risposta di 16 cent, VODAFONE 49 cent di Euro al minuto più scatto alla risposta di 15 cent, WIND 49 cent di Euro al minuto più scatto alla risposta di 15 cent, H3G 49 cent di Euro al minuto più scatto alla risposta di 16 cent. Il servizio ha una durata massima da rete fissa di 125 minuti e per un costo massimo di 15 euro. Il servizio è destinato ai maggiorenni ed è erogato dal San Raffaele spa.

PIS 12000000039.001
