# Supplementary material for: Acute Modulation of Brain Connectivity in Parkinson Disease after Automatic Mechanical Peripheral Stimulation: A Pilot Study
Source: PLoS One. 2015 Oct 15;10(10):e0137977. doi: 10.1371/journal.pone.0137977 (PMC4607499; doi:10.1371/journal.pone.0137977)
Supplement: S2 Protocol — (PDF) [file pone.0137977.s005.pdf]

Translation of the approval of the Clinical Study by the competent Ethical Committee

IRCCS San Raffaele Pisana, Rome, Medical Ethics Committee

Constituted according to Italian Ministerial Decree, dated May 12th, 2006

Minutes nr. 6/2013 – Meeting of July 22nd, 2013

Postponed to September 2nd, 2013

At 3.00 p.m. starts the meeting of the Ethics Committee (EC) composed by:

**Nominativo / qualifica-disciplina**	**Struttura di appartenenza**
Prof. Paolo Arbarello / Medico Legale	Membro esterno
Prof. Carlo Bertolini / Clinico	Membro esterno
Prof. Giovanni Capelli / Biostatistico	Membro esterno
Dott.ssa Rosamaria Adorisio / Farmacista	IRCCS San Raffaele Pisana
Dott. Adolfo Teobaldo De Girolamo / Esperto Materia Giuridica	Membro esterno
Dott.ssa Maria Fattori / Settore infermieristico	San Raffaele SpA
Prof. Massimo Fini / Direttore Scientifico	IRCCS San Raffaele Pisana
Dott. Francesco Frascà / Medico Medicina Generale territoriale	Membro esterno
Prof. Davide Lauro / Presidente	Membro esterno
Prof. Vincenzo Mollace / Farmacologo	IRCCS San Raffaele Pisana
Dott. Graziano Onder / Clinico	Membro esterno
Dott.ssa Isabella Maria Lucrezia Richichi / Direttore Sanitario	IRCCS San Raffaele Pisana
Dott.ssa Rita Salotti / Farmacista	Membro esterno
Don Antonino Sapuppo / Esperto Bioetica	Membro esterno
Sig.ra Lynda Ann Vitali Johnson / Rappresentante del volontariato per l'assistenza/associazionismo di tutela dei pazienti	Membro esterno
Dott.ssa Fabiola D'Angeli / Segretario (non partecipante al voto)	IRCCS San Raffaele Pisana
Selvarolo Rosaria / Segreteria Comitato Etico (non partecipante al voto)	IRCCS San Raffaele Pisana

Assenti: Dott.ssa Adorisio, Prof. Paolo Arbarello, Dott. Cesario, Dott. Frascà, Prof. Vincenzo Mollace, Dott. Onder, Dott.ssa D'Angeli

Minutes prepared by: R. Selvarolo

The President of the EC, having verified that the legal number of participants is present, declares open the meeting.

The Committee discusses the following agenda:

OMISSIS

Request of Parere Unico (Opinion on the Study)
Book of Opinions 12.13

Clinical Study conducted with the medical device code "GONDOLA PILOTA" (*Gondola Pilot study)*

Cross-over Pilot Study on the use of the "Gondola" device for the motor rehabilitation of patients affected by Parkinson's disease and by Progressive Supranuclear Palsy

SPONSOR: San Raffaele Pisana
Research area: Parkinson
Principal investigator: Fabrizio Stocchi

The following documentation has been analyzed:

- Cover letter for the transmission to the EC, dated 20/05/2013
- Synopsis, dated 20/05/2013, rev. 1
- Full project, dated 20/05/2013, rev. 1
- Flow-Chart, dated 20/05/2013 rev. 1
- List of participant centers, dated 20/05/2013 rev. 1
- Zero indemnity declaration, dated 20/05/2013 rev. 1
- List of personnel, dated 20/05/2013 rev. 1
- Curricula vitae of participants
- Declaration regarding conflicts of interest, dated 20/05/2013 rev. 1
- Principal investigator's declaration related to a Clinical Trial with a no Profit promoter, dated 20/05/2013
- Information note-Informed consent, dated 20/05/2013 rev. 1
- Information note-Informed consent for the Legal Guardian/Caregiver, dated 20/05/2013 rev. 1
- Information letter to the General Practitioner, dated 20/05/2013 rev. 1
- Insurance certificate and Insurance policy on Device, valid from April 1st, 2013 through Dec. 31st, 2016
- GONDOLA Technical File, Rev. 03 28/05/2013
- GONDOLA Instruction Manual
- Risk assessment GONDOLA Rev. 0
- Agreement of device free loan for use and Gondola CE Certification, letter dated 05/03/2013
- CRF (*Case Report Form*), dated 20/05/2012 rev. 1

The President describes the clinical study.

The Ethical Committee expresses the opinion that:
1. the study respects the Ethical and Scientific Criteria that allow and justify the study;
2. the expected benefits, both at therapeutic level and at public health level, do justify the risks implicit in the study;

3. the documents to provide information to the patient and to gather the patient's consent are correct and functional to the goal;
4. the right to physical and mental integrity and to the privacy of the involved subjects, and the right to personal data protection, is defended in compliance with the requests of Italian Legislative Decree nr. 196/2003, and with its addenda and modifications;
5. the protection of personal rights, of the safety and of well-being of the subjects included in the clinical study prevails on the scientific interests and on the interest of the society;
6. the study is designed and will be managed in compliance with the Helsinki declaration and with amendments to the ethical principles, and of good clinical practice.

Taken into consideration all the above, the Ethical Committee expresses Positive Opinion on the Clinical Study (Parere Unico).

The Ethical Committee highlights what follows:
a) The Ethical Committee reminds to the promoter of the study to respect the obligations to:

- ✓ notify the Clinical Study to the Italian Ministry of Health (in conformity to the requirements of the Ministerial Decree of the Ministry of Health dated August 2nd, 2005 – published on the "Gazzetta Ufficiale" nr. 210 dated September 9th, 2005);
- ✓ provide the Ethical Committee with such notification to the Italian Ministry of Health;
- ✓ provide the Ethical Committee with possible opinions expressed by the Ministry of Health in relation to the Clinical Study, if any;
- ✓ provide the Ministry of Health and the competent Ethical Committees with the final report at the end of the Clinical Study, which shall include a review of all data gathered during the study. The final report shall be prepared in compliance with the EN ISO 14155-1:2003 norm and with its updates, or in compliance with other analogue norms applicable at international level, that must be cited in the report.

The request of administrative authorization – needed to start the Clinical Study – will be issued upon duly notification of the study to the Ministry of Health.

The Ethical Committee asks to be notified when the study activities are initiated, upon completion of such activities, and shall be notified of the reasons that caused the interruption, shall this happen. The Ethical Committee shall also receive the Study advancement reports and the final Study report. Should adverse events happen, they need to be notified to the EC in a timely manner. The Study shall be conducted in strict compliance with all applicable regulations. It is also important that all Study documentation (including data forms, and specifically the informed consent forms) shall be filed and kept under the responsibility of the Investigators for no less than 7 years after the completion of the study. The documentation shall be easily retrievable and shall be made available to the competent authorities in case of controls (i.e. Ministry of Health).

Rome, September 2nd, 2013

Signed: The President of the Ethical Committee, Prof. Davide Lauro

Signatures of members who were present to the meeting:

Riunione Comitato Etico IRCCS San Raffaele Pisana
02 Settembre 2013

NOMINATIVO MEMBRI:	SPECIFICA	FIRMA:
Adorisio Dott.ssa Rosamaria	Farmacista ex officio	ASSENTE
Arbarello Prof. Paolo	Esperto Medicina Legale	
Bertolini Prof. Carlo	Clinico	
Capelli Prof. Giovanni	Biostatistico	
De Girolamo Prof. Adolfo T.	Esperto Materia Giuridica	
Fattori Dott.ssa Maria	Settore Infermieristico	
Fini Prof. Massimo ☒	Direttore Scientifico ex officio	
Cesario Dott. Alfredo ☐	Sostituto permanente	ASSENTE
Frascà Dott. Francesco	Medico Medicina Generale territoriale	ASSENTE
Lauro Prof. Davide	Presidente	
Mollace Prof. Vincenzo	Farmacologo	ASSENTE
Onder Dott. Graziano	Clinico	ASSENTE
Richichi Dott.ssa Isabella M.L.	Direttore Sanitario ex officio	
Salotti Dott.ssa Rita	Farmacista	
Sapuppo Don Antonino	Esperto Bioetica	
Vitali Johnson Sig.ra Lynda Ann	Rappresentante del volontariato per l'assistenza/associazionismo di tutela dei pazienti	
D'Angeli Dott.ssa Fabiola	Segretario Comitato Etico	ASSENTE
Selvarolo Rosaria	Segreteria Comitato Etico	
