# Supplementary material for: Acute Modulation of Brain Connectivity in Parkinson Disease after Automatic Mechanical Peripheral Stimulation: A Pilot Study
Source: PLoS One. 2015 Oct 15;10(10):e0137977. doi: 10.1371/journal.pone.0137977 (PMC4607499; doi:10.1371/journal.pone.0137977)
Supplement: S1 Results — (DOC) [file pone.0137977.s006.doc]

**S_Results**

**Acute modulation of brain connectivity in Parkinson disease after Automatic Mechanical Peripheral Stimulation: a pilot study**

**Changes of Functional Connectivity in the Resting State (without inclusion of 6 head motion parameters as regressors)**

One sample t-test analyses showed significant (p<0.05, FEW corrected) differences of RSFC with the selected seed regions following effective AMPS but not on sham conditions. Group differences were always oriented toward a significant increase of connectivity. Reduction of connectivity was not found with any of the seed regions. The primary sensory-motor cortex showed significantly stronger connectivity with the left superior parietal lobule, the left lateral occipital cortex and the cerebellar right crus II (S_Table 1 and S_Figure 1). The second higher level analysis to test the specific changes induced by the effective stimulation after ruling out sub-threshold effects of sham stimulation showed significantly higher connectivity with the left and right mesial post-central and pre-central gyrus (i.e. intraregional connectivity) and with the right and left precuneus cortex (S_Table 2 and S_Figure 2).

The supplementary motor area showed significant increase of connectivity with the left superior parietal lobule and the left lateral occipital cortex (S_Table 1 and S_Figure 1). The second higher-level analysis did not show significant changes of connectivity with the supplementary motor area.

The nucleus striatum demonstrated significant increase of connectivity with two brain areas that included the right lateral occipital cortex (inferior portion), the right inferior temporal cortex (temporo-occipital portion), the right temporal and occipital fusiform gyrus, the right parahyppocampal gyrus and the right middle temporal gyrus (posterior portion) after effective but not after sham stimulation (S_Table 1 and S_Figure 1). The second higher level analysis did not show significant changes of connectivity with the nucleus striatum. Globi pallidi and thalami did not demonstrate above threshold changes of functional connectivity both on effective and sham conditions.

The cerebellum showed significantly stronger connectivity with the left paramedian cerebellar cortex (i.e. intraregional connectivity), lobules I to VI, and with the right lateral occipital cortex and the right fusiform gyrus (occipital cortex) (S_Table 1 and S_Figure 1).

The second higher level analysis showed significantly higher connectivity induced by the effective stimulation with the left paramedian cerebellar cortex (i.e. intraregional connectivity, lobules VIII to X), the left lateral occipital cortex and the left precuneus and cuneal cortex (S_Table 2 and S_Figure 2).

**S_Table 1.** **Local maxima in clusters of significantly (p < 0.05, FWE corrected) higher resting-state temporal correlation with the BOLD signal of the seed-ROIs after one session of AMPS.**

| Seed ROI | Cluster location | Cluster size (voxels) | MNI x | MNI y | MNI z | Z score |
| --- | --- | --- | --- | --- | --- | --- |
| Primary Sensory-Motor Cortex | Left Superior Parietal Lobule | 783 | -26 | -54 | 48 | 3.53 |
|  | Left Lateral Occipital Cortex |  | -20 | -68 | 52 | 3.20 |
|  | Cerebellum Right Crus II | 480 | 26 | -84 | -40 | 3.46 |
| Supplementary Motor Area | Left Superior Parietal Lobule | 523 | -28 | -58 | 54 | 3.03 |
|  | Left Lateral Occipital Cortex |  | -30 | -62 | 60 | 3.00 |
| Nucleus Striatus | Right Lateral Occipital Cortex, inferior | 810 | 56 | -62 | -4 | 3.45 |
|  | Right Inferior Temporal Gyrus, temporo-occipital |  | 46 | -58 | -12 | 3.1 |
|  | Right Occipital Fusiform Gyrus |  | 28 | -74 | -14 | 3.33 |
|  | Right Temporal Pole | 605 | 40 | 14 | -24 | 3.24 |
|  | Right Parahyppocampal gyrus/temporal fusiform cortex |  | 28 | -26 | -28 | 3.17 |
|  | Right Middle Temporal gyrus, posterior |  | 46 | -24 | -8 | 2.82 |
|  | Right Temporal fusiform cortex |  | 34 | -34 | -24 | 2.77 |
| Cerebellum | Right Lateral Occipital Cortex | 1133 | 52 | -64 | -6 | 3.95 |
|  | Right Occipital fusiform gyrus |  | 22 | -88 | -4 | 3.70 |
|  | Left Cerebellum, I-IV lobules | 576 | -6 | -50 | -22 | 3.02 |
|  | Left Cerebellum VI lobule |  | -20 | -58 | -22 | 2.86 |
|  | Left Cerebellum, V lobule |  | -4 | -58 | -20 | 2.86 |

**S_Table 2.** **Local maxima in clusters of significantly (p < 0.05, FWE corrected) stronger connectivity in the effective AMPS vs. sham stimulation.**

| Seed ROI | Cluster location | Cluster size (voxels) | MNI x | MNI y | MNI z | Z score |
| --- | --- | --- | --- | --- | --- | --- |
| Sensory-Motor Cortex | Left and Right Postcentral Gyrus, Precentral Gyrus and Precuneus Cortex | 403 | -4 | -38 | 56 | 3.50 |
| Cerebellum | Left Lateral Occipital Cortex, superior | 628 | -38 | -78 | 16 | 3.64 |
|  | Left precuneus and cuneal cortex |  | -10 | -68 | 22 | 3.00 |
|  | Left Cerebellum, IX lobule | 571 | -12 | -52 | -34 | 3.28 |
|  | Left Cerebellum, X lobule |  | -24 | -40 | -42 | 3.26 |
|  | Left Cerebellum, VIIIa lobule |  | -26 | -48 | -46 | 2.93 |

**S_Figure Legends**

**S_Figure 1:** Z-statistic images showing clusters of significantly increased RSFC (p < 0.05, cluster-level FWE corrected) after one session of effective plantar mechanical stimulation of the primary sensory motor cortex (a), the supplementary motor area (b), the nucleus striatum (c) and the cerebellum (d), overlaid onto a MNI-registered anatomical 3D-T1 volume. Seed regions of interest are red-coloured in the panels on the left. MNI coordinates (x, y, z) of the maximal Z-scores are presented in Table 2. Images follow the radiological convention.

**S_Figure 2:** Z-statistic images showing clusters of significantly increased RSFC (p < 0.05, cluster-level FWE corrected) in the effective vs. sham plantar mechanical stimulation for the sensory motor cortex (a) and the cerebellum (b). Seed regions of interest are red-coloured in the panels on the left. MNI coordinates (x, y, z) of the maximal Z-scores are presented in Table 3. Images follow the radiological convention.

**S_Figure 1**


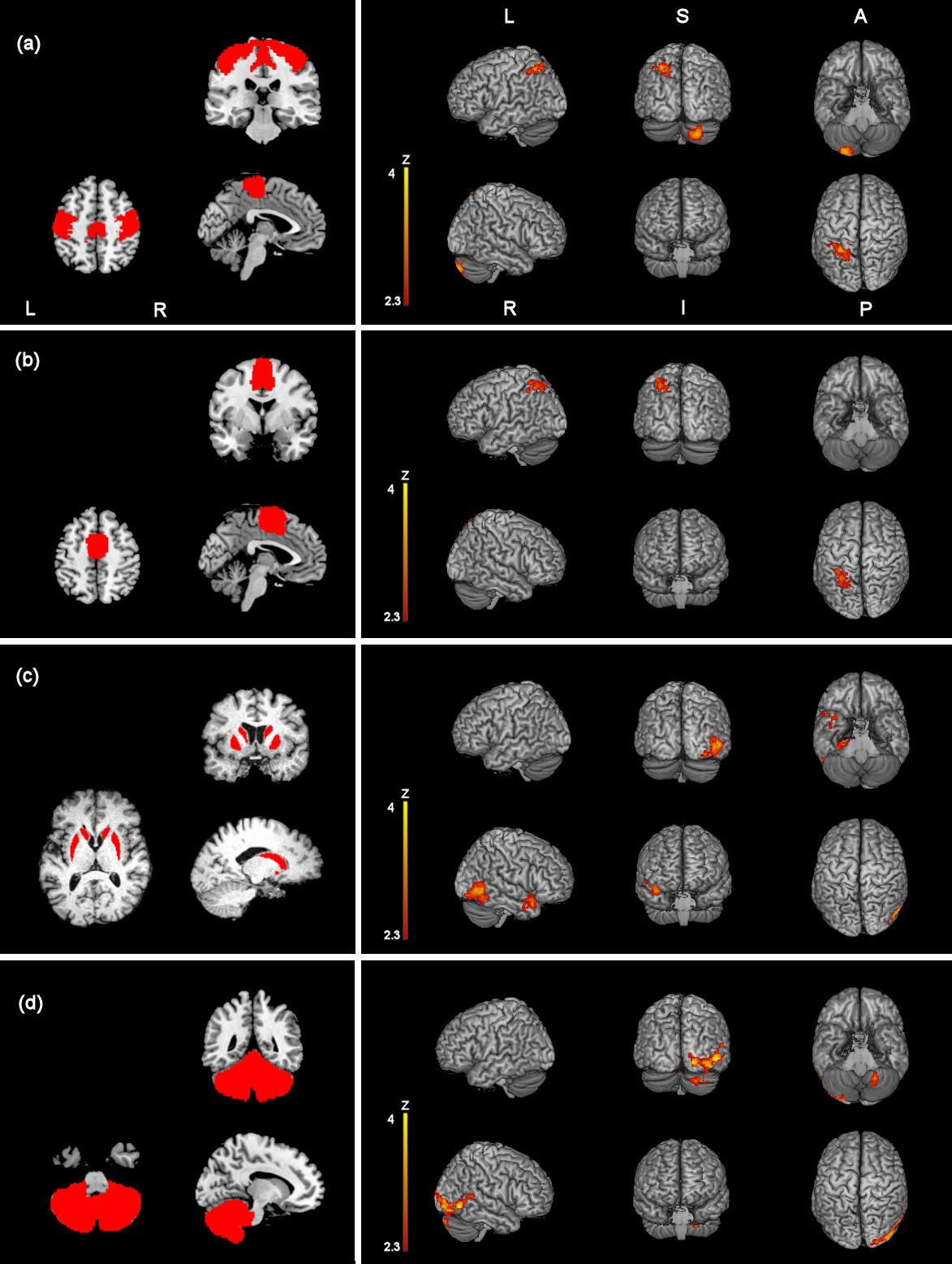


**S_Figure 2**

**
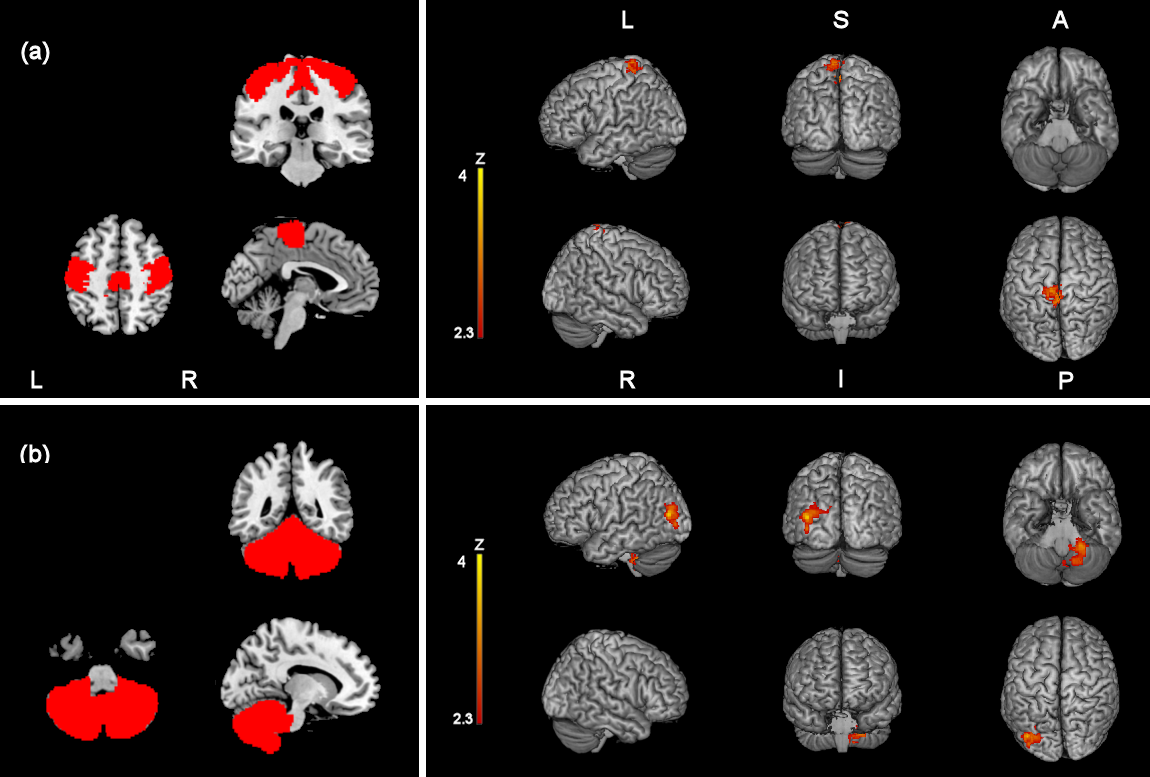
**
